# Understanding the Role of Terrestrial and Marine Carbon in the Mid‐Latitude Fjords of Scotland

**DOI:** 10.1029/2022GB007434

**Published:** 2022-11-11

**Authors:** C. Smeaton, W. E. N. Austin

**Affiliations:** ^1^ School of Geography & Sustainable Development University of St Andrews St Andrews UK; ^2^ Scottish Association for Marine Science Oban UK

**Keywords:** carbon, fjords, terrestrial, sediment, marine, mid‐latitude

## Abstract

The sediments within fjords are critical components of the mid‐ to high‐latitude coastal carbon (C) cycle, trapping and storing more organic carbon (OC) per unit area than other marine sedimentary environments. Located at the land‐ocean transition, fjord sediments receive OC from both marine and terrestrial environments; globally, it has been estimated that 55%–62% of the OC held within modern fjord sediments originates from terrestrial environments. However, the mid‐latitude fjords of the Northern Hemisphere have largely been omitted from these global compilations. Here we investigate the mechanism driving the distribution of OC originating from different sources within the sediments of 38 Scottish fjords. From an array of fjord characteristics, the tidal range and outer sill depth were identified as the main drivers governing the proportions of marine and terrestrial OC in the sediments. Utilizing this relationship, we estimate that on average 52% ± 10% of the OC held within the sediments of all Scotland's fjords is terrestrial in origin. These findings show that the Scottish fjords hold equivalent quantities of terrestrial OC as other global fjord systems. However, the analysis also highlights that the sediments within 29% of Scottish fjords are dominated by marine derived OC, which is driven by local fjord geomorphology and oceanography.

## Key Points

- 28%–72% of the organic carbon (OC) held within the surficial sediments of the mid‐latitude fjords of Scotland originates from terrestrial sources
- Annually, the sediments within Scottish fjords store between 8% and 10% of the OC lost from their catchments due to soil erosion
- Local geomorphology and oceanography drive the distribution of both terrestrial and marine derived OC in Scotland's fjord sediments

## 1 Introduction

The burial of organic carbon (OC) within continental margins accounts for over 90% of the OC trapped in the marine environment each year (Berner, 1982; Hedges & Keil, 1995). Recently, coastal sediments (Bauer et al., 2013; Bianchi et al., 2018) and fjords in particular have been recognized as important hotspots for the burial (Smith et al., 2015) and long‐term storage of OC (Smeaton et al., 2017). Globally, it is estimated that 18 Mt of OC is buried in fjord sediments annually, which equates to 11% of the total oceanic OC burial (Smith et al., 2015). Key to fjords ability to trap and store OC is geomorphology; typically, over‐deepened fjord basins facilitate rapid sedimentation of organic matter which is quickly incorporated into the seabed sediments (Bianchi et al., 2020; Syvitski & Shaw, 1995). Additionally, the transfer of terrestrially derived OC (OC_terr_) from adjacent catchments to the marine sediment is believed to be significant component and a major driver of OC burial in fjords both today (González et al., 2019; Hinojosa et al., 2014; Sepúlveda et al., 2011; Smith et al., 2010) and throughout the Holocene (Nørgaard‐pedersen et al., 2006; Smeaton, Cui, et al., 2021). Current estimates suggests that 55%–62% of the OC buried in fjord sediments each year is derived from the terrestrial environment (Cui, Bianchi, Savage, & Smith, 2016).

The current global estimate of the quantity of OC_terr_ held within fjord sediments is largely based on data from the Southern Hemisphere and more precisely New Zealand (Cui, Bianchi, Savage, & Smith, 2016; Smith et al., 2015), where the fjords tend to be more restricted (i.e., land‐locked) and have anoxic and hypoxic bottom waters (Cui, Bianchi, Savage, & Smith, 2016; Hinojosa et al., 2014; Smith et al., 2010). Low oxygen conditions do exist in Northern Hemisphere fjords but they are far from common (Friedrich et al., 2014; Gillibrand et al., 1995). Northern Hemisphere systems tend to have deeper submarine sills and are less geomorphologically restricted (Bianchi et al., 2020; Howe et al., 2010; Smeaton et al., 2017; Syvitski & Shaw, 1995) resulting in well‐ventilated oxygenated bottom waters (Austin & Inall, 2002; Gillibrand et al., 1995). The relatively low proportion of OC_terr_ observed in the sediments of these less restricted exchange systems suggest that marine derived OC (OC_mar_) may play a more significant role in driving OC burial and storage (Faust & Knies, 2019; Smeaton & Austin, 2017) which potentially means that existing global compilations of data may overestimate the quantity and role of OC_terr_ in driving OC burial and storage in fjords globally.

The mid‐latitude fjords of Scotland have been shown to store 252 ± 62 Mt of OC in their post‐glacial sediments (Smeaton et al., 2017). Today these modern sediments bury 84,000 tonnes of OC annually (Smeaton, Hunt, et al., 2021). Yet our understanding of the relative contribution of the sources of the OC held within these sizable OC stores is lacking. The limited available data estimates that 24%–45% of the OC held within the surficial sediments originates from the terrestrial environment (Smeaton & Austin, 2017), which is notably lower than current global estimates (Cui, Bianchi, Savage, & Smith, 2016), suggesting a larger marine influence across these Scottish systems.

Here we quantify the OC_terr_ fraction within the sediments of a range of Scottish fjords with differing catchment and geomorphological characteristics to both quantify the OC_terr_ held within the sediments and in combination with existing data understand the mechanisms that govern the distribution, burial and storage of OC_terr_ in these fjords. By understanding these characteristics there is potential to upscale the OC_terr_ estimates to all Scottish fjords, allowing a greater exploration of the relationship between the terrestrial and marine environment at a national scale.

## 2 Study Area

**Figure 1:**
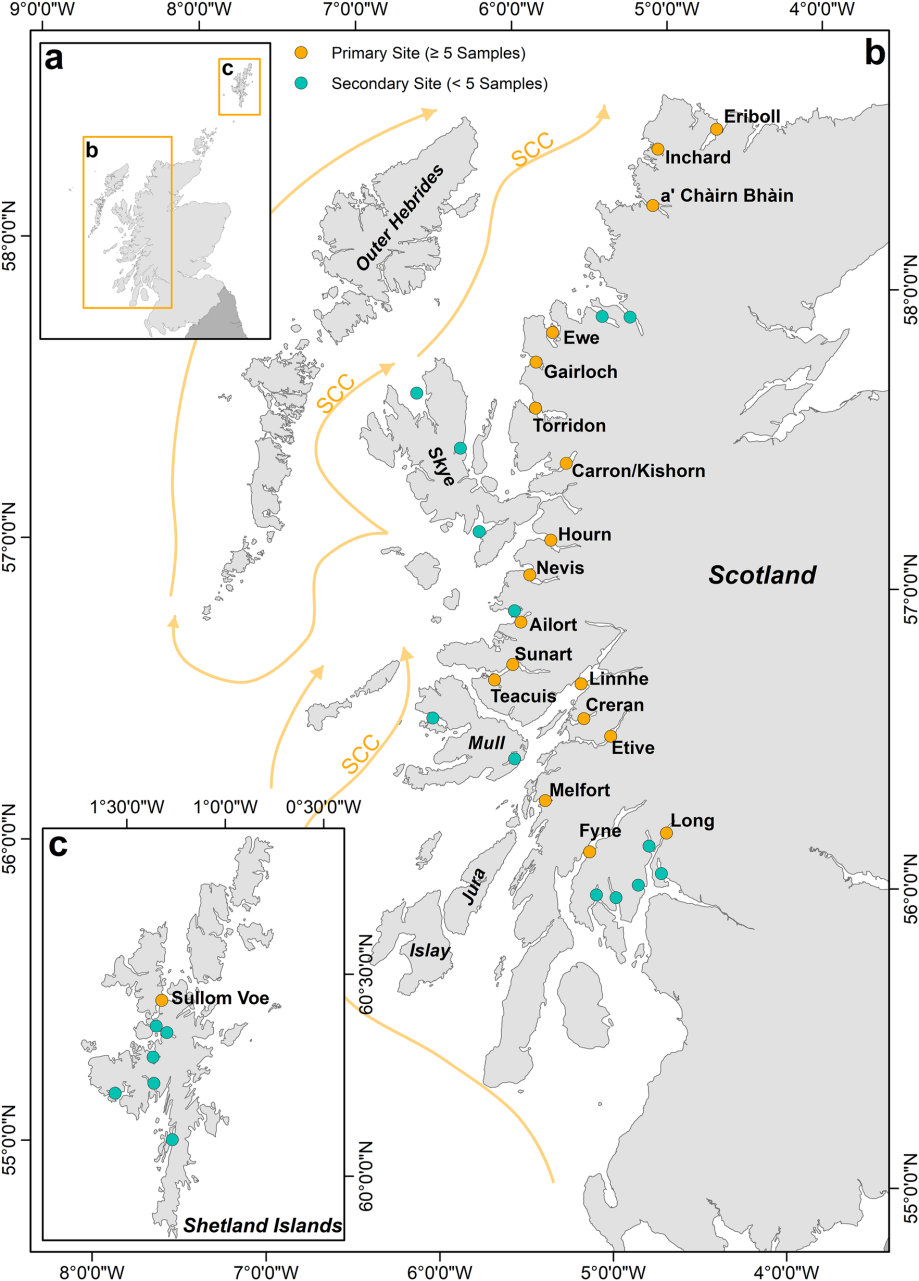
Location of fjords sampled as part of this study. (a) Overview map illustrating the location of the fjords in relation to Scotland; (b) Mainland and inner hebrides (Skye and Mull) sampling sites; (c) sampling sites within the waters of the Shetland Islands. Orange symbols represents fjords with ≥5 sampling sites, blue symbols correspond to systems with <5 sampling sites. Arrows illustrate the primary oceanographic currents within the area. Detailed maps of sampling sites within each fjord can be viewed in Figures S2–S5 in Supporting Information S1.

The fjords situated on the west coast of Scotland total 226 individual systems (Figure 1) split between 111 large fjords (over 2 km long, with fjord length at least twice fjord width) (Edwards & Sharples, 1986) and 115 smaller systems (Smeaton & Austin, 2019). In total the fjords occupy an area of 2,608 km^2^ (Smeaton, Yang, & Austin, 2021) and drain 14,424 km^2^ (∼18% of mainland Scotland) of some of the most C rich terrestrial environments in Europe (Lilly & Donnelly, 2012) (Figure S1 in Supporting Information S1). The Scottish fjords have similar features to other temperate vegetated fjords found in Norway, New Zealand, Chile and North America (Bianchi et al., 2020; Syvitski & Shaw, 1995). Previous studies have shown that Scotland's fjords can be characterized into several groups based on their glacial history and resultant geomorphology (Smeaton et al., 2017). The mainland and Inner Hebrides (Islands of Skye, Mull, Jura and Isaly) systems are classic fjords with over‐deepened basins (Howe et al., 2002; Syvitski & Shaw, 1995). With the exception of Sullom Voe (Figure 1c) the Outer Hebrides and the Shetland Island systems are shallower with a more subdued submarine geomorphology and are referred to as fjards. Fjards are also products of glacial processes but unlike fjords they are relatively shallow and flat‐bottomed and lack the geomorphological characteristics (i.e., sills) which result in increased sediment accumulation rates observed in fjords (Syvitski et al., 1987). These regional differences in geomorphology result in the mainland fjords being the main repository (94%) for the 252 ± 62 Mt of OC that is stored in the postglacial sediments of Scotland's fjords (Smeaton et al., 2017).

The coastal waters of western Scotland are heavily influenced by the Scottish Coastal Current (SCC) (Ellett, 1979; Ellett & Edwards, 1983), which flows northwards along the Atlantic coast of Scotland (Figure 1). In comparison to other regional oceanographic currents, such as the North Atlantic Current (NAC), the SCC is colder and fresher (Inall et al., 2009). Occasional incursions of saline (>35) water from the North Atlantic occurs north of Ireland which mixes with the SCC (McKay et al., 1986) creating an area of high productivity (Ellett, 1979). Unlike the fjords of New Zealand, where hypoxic and anoxic conditions are common (Cui, Bianchi, Savage, & Smith, 2016; Hinojosa et al., 2014), the mid‐latitude fjords of Scotland tend to be well ventilated with oxygenated bottom waters (Austin & Inall, 2002; Gillibrand & Amundrud, 2007; Gillibrand et al., 1995, 2005) which is largely driven by local oceanographic conditions such as tidal mixing combined with coastal/fjord density differences and the submarine geomorphology of the fjord themselves (McIntyre & Howe, 2010; Smeaton et al., 2017).

## 3 Materials and Methods

### 3.1 Sampling

Surficial sediment samples were collected from 36 fjords across the mainland and islands of Scotland (Figure 1) supplementing data from two other sites (Loch Sunart and Teacuis) previously sampled (Smeaton & Austin, 2017). In total, 450 samples were collected between 2016 and 2020, with the majority of samples being collected from onboard the RV *Seòl Mara*, RV *Calanus*, MRV *Alba na Mara* and the MRV *Scotia*. Samples were collected using a grab sampler (Day and Van‐Veen grabs) along transects from the head to the mouth of each of the fjords (Figures S2–S5 in Supporting Information S1). The Day grab provides a platform to collect undisturbed samples allowing the uppermost layers of sediment to be collected (0–2 cm). The Van‐Veen grab provides a composite sample that represent the upper 10 cm of sediment.

The 38 fjords for which we have samples can be classed as primary or secondary sites based on the number of samples, where primary sites have ≥5 samples (*n* = 19) and secondary sites have <5 samples (*n* = 19). At each sampling site the location and water depth were recorded alongside the sediment type which was classified following the Folk (1954) scheme. Samples were frozen on‐board each vessel before being returned to the University of St Andrews. Full details of the sample collection sites, sampling method and research vessels can be found in Supporting Information S1.

### 3.2 Bulk Elemental and Stable Isotope Analysis

Samples were freeze dried and milled to a fine powder; approximately 12 mg of milled sediment was placed into tin capsules and sealed, and a further 12 mg was placed into silver capsules. The samples encapsulated in silver underwent acid fumigation (Harris et al., 2001) to remove carbonate (CaCO_3_) and were dried for 24 hr at 40°C. Stable isotope analysis was undertaken at the NERC Life Science Mass Spectrometry Facility (NERC LSMSF) using an elemental analyzer coupled to an isotope ratio mass spectrometer. The acidified samples were analyzed for OC and ẟ^13^C_org,_ while nitrogen (N) and ẟ^15^N values were produced from the tin samples. By analyzing the N and δ^15^N separately, we reduce the potential risk of altering these values through exposure to acid (Kennedy et al., 2005). Triplicate measurements of samples (*n* = 90) produced standard deviations (1σ) of 0.02% for N and 0.08‰ for δ^15^N, 0.05% for OC and 0.07‰ for δ^13^C_org_. Further quality control was assured by repeat analysis of high OC sediment standard (B2151) with reference values for C of 7.45% ± 0.14%, ẟ^13^C of −28.85 ± 0.10‰, N of 0.52% ± 0.02% and ẟ^15^N of 4.32 ± 0.2‰. The reference standards (*n* = 45) deviated from their known values by: OC = 0.06%, ẟ^13^C = 0.11‰, *N* = 0.03% and ẟ^15^N = 0.11‰. The isotope values are reported in standard delta notation relative to Vienna Peedee belemnite (VPDB) and air. The N/C ratio was reported as molar ratios: N/C = (N/14)/(OC/12).

### 3.3 Fraction of Terrestrial OC

Bulk elemental (N/C) and stable isotope (ẟ^13^C_org_ and ẟ^15^N) values representing the OC sources (e.g., terrestrial and marine) were determined from samples collected from the catchments and foreshore of Scotland reported by Smeaton and Austin (2017) and Smeaton et al. (2022). The terrestrial source values were determined from different soils (*n* = 37) and vegetation (*n* = 37) samples across an array of land uses, while the marine source values were calculated from 57 samples across a range of biota. Weighted means were calculated to assure that no one source of OC (e.g., macro‐algae) was over represented in the end‐member values. The terrestrial source values were calculated assuming equal contributions of OC from the soil and terrestrial vegetation; while the marine end‐member values were calculated assuming equal contributions of OC from macro‐algae, phytoplankton and zooplankton.

Recent work within Loch Sunart indicated that there is minimal fossil/petrogenic C input (<0.1%) to the sediments (Smeaton, Cui, et al., 2021). The catchment of the fjords in this study are similar to that of Loch Sunart with the bedrock geology largely comprised of metamorphic and igneous rocks (Smith, 2009). Therefore, it is unlikely that fossil/pyrogenic C will influence the ẟ^13^C_org_ values.

A binary (or two end‐member) mixing model based upon Thornton and McManus (1994) was employed to calculate the fraction of terrestrial OC in the sediment (Equations (1), (2), (3)). Within this model ẟ^13^C_org_ values were used as the tracer as it has been shown to be a more reliable and robust tracer of OC source than either N/C or δ^15^N when used alongside a binary mixing model (Faust & Knies, 2019; Hinojosa et al., 2014; Smeaton & Austin, 2017). The binary mixing model was chosen over a Bayesian approach (Fernandes et al., 2014; Smeaton & Austin, 2017) to allow comparisons to global *F*_terr_ estimates calculated using the binary mixing model approach (i.e., Cui, Bianchi, Jaeger, & Smith, 2016; Faust & Knies, 2019; Hinojosa et al., 2014; Silva & Prego, 2002).

(1) $$F_{terr}=\frac{\left(\delta^{13}C_{sample}-\delta^{13}C_{mar}\right)}{\left(\delta^{13}C_{terr}-\delta^{13}C_{mar}\right)}$$

(2) $$F_{terr}+F_{mar}=1$$

(3) $${\%OC}_{terr}=F_{terr}\times \%OC$$

The binary mixing model calculations were carried out in combination with Markov Chain Monte Carlo (MCMC) simulations in the OpenBUGS software (Lunn et al., 2009). In the simulations, 100,000 out of 1,000,000 random samples from a normal distribution of each end‐member were taken to populate the mixing models calculation steps (Equations (1), (2), (3)). This results in a significant number of solutions being generated for each sample. The mean, range and standard deviation of the *F*_terr_ were calculated for each sample from the MCMC solutions.

To estimate the fraction of terrestrial OC held within the surficial sediment of each fjord distance‐weighted means were calculated. The distance‐weighted mean is a measure of central tendency, where weighting coefficient for each data point is determined as the inverse sum of distances between a data point and the other data points (Dodonov & Dodonova, 2011; Machuca‐Mory & Deutsch, 2013). Therefore, central observations in a data set get the highest weights, while values in the tails of a distribution are down weighted.

### 3.4 Quantifying the Relationship Between OC_terr_ and Fjord Characteristics

The quantity of OC_terr_ and OC_mar_ in fjord sediments is largely governed by the inflow of freshwater (OC_terr_) and oceanic waters (OC_mar_). Faust and Knies (2019) conceptualized the burial and storage of OC_terr_ using these variables and were able to categorize North Atlantic fjords into four groups:

1. Low oceanic water inflow and low freshwater inflow: OC_terr_ dominated (>50% OC_terr_)
2. Low oceanic water inflow and high freshwater inflow: OC_terr_ dominated (>50% OC_terr_)
3. High oceanic water inflow and high freshwater inflow: Equal contributions of OC_mar_ and OC_terr_ (50:50, OC_terr_:OC_mar_)
4. High oceanic water inflow and low freshwater inflow: OC_mar_ dominated (>50% OC_mar_)

The submarine geomorphology of the fjord is the main variable which determines the inflow of oceanic water, while rainfall, catchment area and hence runoff determines the freshwater input (Bianchi et al., 2020; Syvitski et al., 1987).

The submarine geomorphology, and in turn fjord exchange and renewal processes, is commonly defined by the depth of the submarine sills (Cuthbertson et al., 2021; Rüggeberg et al., 2011; Smeaton & Austin, 2022). The shallower the sill, the more restricted the system becomes, often reducing oceanic water inflow. Freshwater inflow data to Scottish fjords in the form of typical catchment rainfall (mm yr^−1^) and average runoff data (million m^3^ yr^−1^) is readily available alongside other environmental variables such as sill depth, tidal range, catchment area, fjord volume, mixing depth, flushing time (Edwards & Sharples, 1986). These parameters and combinations thereof, were compared to the quantities of OC_terr_ in the fjords to determine if a statistical relationship exists and if it can be used model the quantity of OC_terr_ stored across the surficial sediments (top 10 cm) of the mid‐latitude fjords of Scotland.

### 3.5 OC Budget for Scottish Mid‐Latitude Fjords

In combination the estimated fraction of OC_terr_, the surficial (top 10 cm) OC stocks (Smeaton & Austin, 2019; Smeaton, Yang, & Austin, 2021), full depth integrated OC stocks (Smeaton et al., 2016, 2017) and burial rates (Smeaton, Hunt, et al., 2021) for each Scottish fjord will facilitate the development of a first order OC budget for the fjord sediments of Scotland.

To better understand the interconnectivity of the catchments and sediments of the Scottish fjords the erosion and loss of terrestrial OC from each catchment will be explored using the Revised Universal Soil Loss Equation (RULSE) model for Europe (Panagos et al., 2015) alongside regional soil OC mapping (Lilly & Donnelly, 2012). The RULSE model produces 100 m^2^ resolution estimates of soil loss rates due to water induced erosion (Panagos et al., 2015). In should be noted that the likelihood of soil erosion due to wind is minimal within these catchments (Borrelli et al., 2017). Erosion rates combined with the soil OC mapping allows C loss rates (tonnes OC m^−2^ yr^−1^) to be calculated and the annual OC loss from these fjord catchments (14,424 km^2^) to be quantified. The total soil eroded in the catchments due to water erosion does not automatically mean that all this material reaches the fjord sediments; water erosion should instead be viewed as a mechanism to mobilize terrestrial OC. The majority of this mobile soil and OC will be redeposited within the catchment in downslope traps or floodplains. Globally, it is estimated that 10% of the soil eroded from the catchment reaches the marine environment (Meade et al., 1990; Stallard, 1998). Despite the semi‐quantitative nature of this framework, it allows an opportunity to make first‐order estimates of the annual OC_terr_ input to these fjords and their role as traps and long‐term stores of OC previously believed to be lost from terrestrial catchments.

## 4 Results and Interpretation

### 4.1 Bulk Elemental and Stable Isotope Analysis

**Figure 2:**
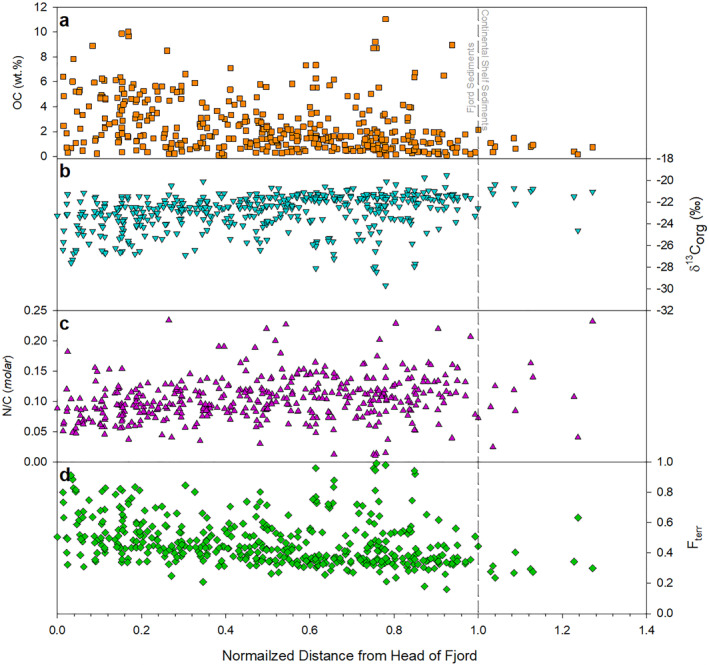
Bulk elemental and isotopic characteristics of the surficial sediments along transects from the head to the mouth (outer sill) of the mid‐latitude fjords of Scotland. (a) OC content (wt. %), (b) δ^13^C_org_ (‰), (c) N/C ratio (*molar*), and (d) the calculated fraction of the OC that originates from the terrestrial environment (*n* = 450). All distances from the head of each fjord have been normalized using the distance between the head of the fjord to the outer sill.

The mean OC content of the surface sediments within Scottish fjords is 2.48% ± 2.19% with values ranging between 0.05% and 11.04%, which generally decrease toward the mouth (outer sill) of the fjords (Figure 2a). The mean δ^13^C_org_ across all systems is −22.9 ± 1.67‰ with values ranging from −19.54‰ to −29.69‰ (Figure 2b), while the δ^15^N values ranges between −0.80 and 10.10‰ with a mean value of 6.17 ± 1.20‰. Additionally, the mean N/C value for the sediment is calculated as 0.10 ± 0.03 with values of between 0.02 and 0.26 (Figure 2; Figure S6 in Supporting Information S1). The fjord sediments display statistically significant differences to the adjacent continental shelf sediments with *p*‐values of 0.011 and 0.005 observed for the OC and δ^13^C_org_ values respectively. The bulk elemental and stable isotope values observed here are comparable to those previously reported from the sediments of Scottish fjords and other vegetated fjords around the world (Table 1).

**Table 1:** Geochemical Characteristics of the Surficial Sediments Held Within the Mid‐Latitude Fjords of Scotland in Comparison With Other Vegetated Fjords

Location	OC (wt. %)	δ^13^C_org_ (‰)	δ^15^N (‰)	Reference
Scotland	2.48 ± 2.19	−29.7–−19.5	−0.80–10.1	This Study
Other Vegetated Fjord Systems				
Scotland	2.88 ± 1.99	−27.7–−17.5	2.36–9.5	Bottrell et al., 2009; Hunt et al., 2020; Loh et al., 2008; Smeaton et al., 2016; Smeaton, Cui, et al., 2021; Smeaton, Hunt, et al., 2021; Smeaton & Austin, 2017
Alaska	5.08 ± 2.44	−22.4–−21.0	—	Cui et al., 2016b; Jaeger et al., 1998; Walinsky et al., 2009
Canada	3.37 ± 2.50	−27.0–−22.6	—	Hage et al., 2022; Ingall et al., 2005; Louchouarn et al., 1997; Nuwer & Keil, 2005; Smith & Walton, 1980; Smittenberg, Hopmans, et al., 2004; Smittenberg, Pancost, et al., 2004; St‐Onge & Hillaire‐Marcel, 2001
Chile	1.79 ± 0.76	−29.0–−19.1	1.3–9.0	Bertrand et al., 2012; Mayr et al., 2014; Rebolledo et al., 2019; Ríos et al., 2020; Ruiz‐Ruiz et al., 2021; Sepúlveda et al., 2005, 2011; Silva et al., 2011; Silva & Prego, 2002
Norway	8.82 ± 4.20	−23.8–−20.9	4.69–6.9	Duffield et al., 2017; Faust et al., 2014; Faust & Knies, 2019; Huguet et al., 2007; Müller, 2001; Skei, 1983; Smittenberg, Hopmans, et al., 2004; Smittenberg, Pancost, et al., 2004; Smittenberg et al., 2005; Velinsky & Fogel, 1999
New Zealand	3.74 ± 1.86	−28.2–−19.1	4.55 ± 2.59	Cui, Bianchi, Savage, & Smith, 2016; Hinojosa et al., 2014; Knudson et al., 2011; Ramirez et al., 2016; Smith et al., 2010
Sweden	4.20 ± 2.54	—	—	Nordberg et al., 2001, 2009

*Note*. Updated from Bianchi et al. (2020) and Cui, Bianchi, Savage, and Smith (2016).

### 4.2 Quantifying Terrestrial and Marine Derived OC

**Figure 3:**
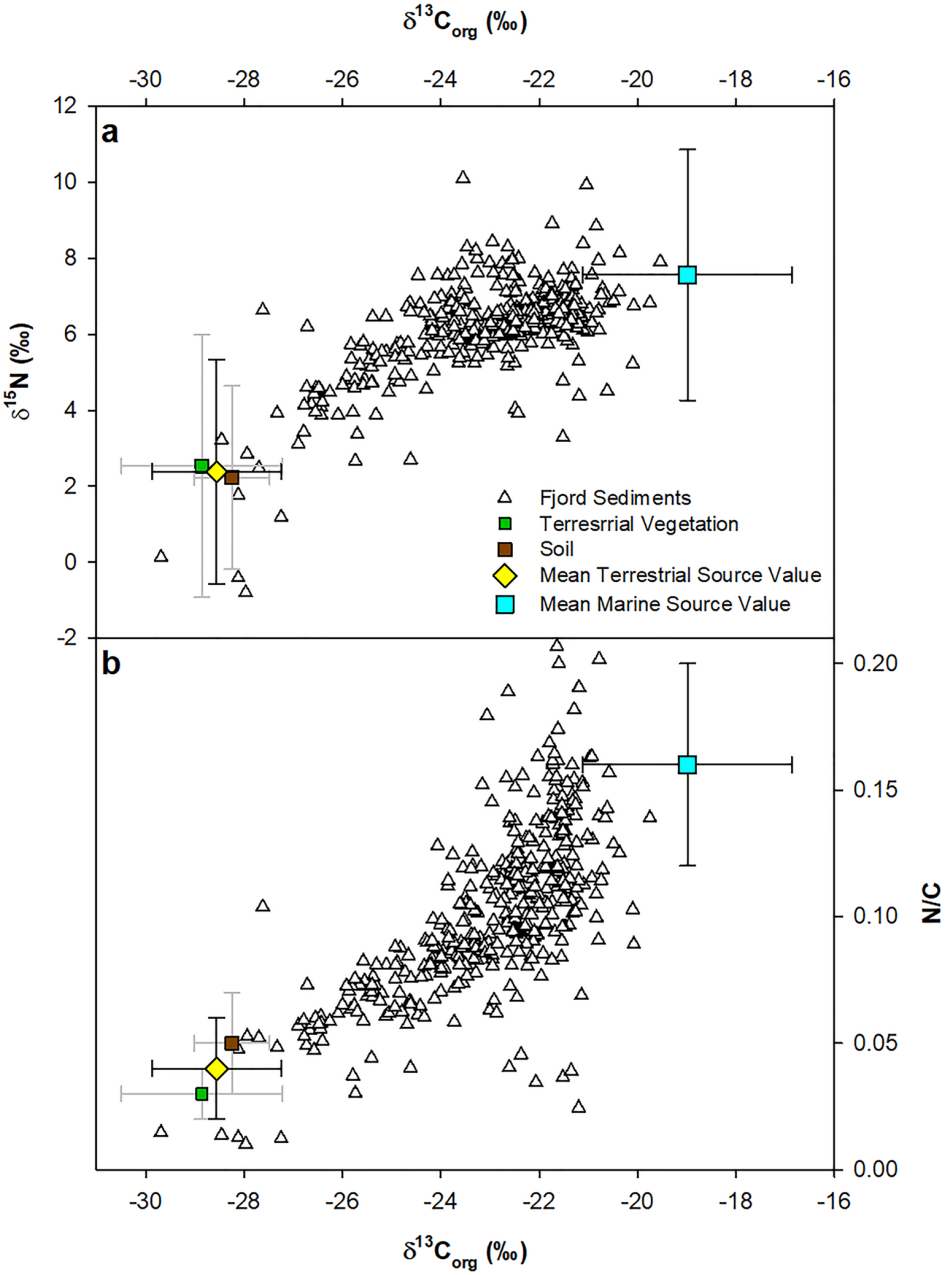
Comparison of bulk elemental and stable isotope values of surficial (top 10 cm) Scottish fjordic sediments with values from the terrestrial and marine environments (Table 2). Cross plots, (a) δ^13^C_org_ versus δ^15^N and (b) δ^13^C_org_ versus N/C in surface sediment of Scotland's fjords.

OC derived from the terrestrial and marine environments was characterized and assigned a δ^13^C_org_, δ^15^N, and N/C values (Table 2) to allow the contribution from each source (terrestrial vs. marine) to be visualized (Figure 3). The source values from the marine and terrestrial environments correspond well to published studies (e.g., Cloern et al., 2002; Cui, Bianchi, Savage, & Smith, 2016; Hinojosa et al., 2014; Thornton & McManus, 1994).

**Table 2:** OC Sources and Their End‐Member Values Used to Visualize the Source of OC Found in the Sediments (Figure 3)

Source	δ^13^C_org_ (‰)	δ^15^N (‰)	N/C (*molar*)
Terrestrial vegetation	−28.86 ± 1.64	2.53 ± 3.45	0.03 ± 0.01
Soil	−28.25 ± 0.77	2.23 ± 2.41	0.05 ± 0.02
Mean terrestrial	−28.56 ± 1.31	2.38 ± 2.96	0.04 ± 0.02
Marine	−18.99 ± 2.13	7.56 ± 3.30	0.16 ± 0.04

*Note*. Smeaton et al. (2022) details the full data set. Full breakdown of the data used to calculate the terrestrial and marine end‐members can be found in Supporting Information S1.

When the bulk elemental and stable isotopes values from the surface sediment are compared with values from the terrestrial and marine environment it becomes apparent that the OC held within the sediments originates from both (Figure 3). The contribution of OC_terr_ and OC_mar_ to the sediments varies significantly across the fjords sampled.

Fjord sediments and the OC held within those sediments are known be highly heterogenic (Smeaton & Austin, 2019). Therefore, it might be expected that the OC contribution from the different sources (terrestrial vs. marine) will also be highly variable. Across the 450 samples, the mean *F*_terr_ value estimated from the δ^13^C_org_ binary mixing model is 0.47 ± 0.21. Across the fjords there is a trend toward lower *F*_terr_ values at the mouth of each fjord (Figure 2d) driven by the increased distance between depositional site and terrestrial OC sources (e.g., larger rivers at the head of the fjord). When each fjord is examined individually there are characteristic differences in *F*_terr_ values moving from the head to the mouth of the fjord (Figure S7 in Supporting Information S1).

Within multi‐basined fjords, there are also distinct differences in *F*_terr_. This is most pronounced in Loch Etive, where the mean *F*_terr_ value of the inner basin is 0.65 ± 0.16 compared to 0.40 ± 0.14 for the outer basin. The difference between the two basins of Loch Etive is extreme in comparison to the other systems (Table S1, Figure S8 in Supporting Information S1). This is driven by three factors; (a) the inner basin drains part of a blanket peatland (Rannoch Moor). (b) The highly restricted inner basin of Loch Etive (Howe et al., 2002) experiences sustained periods of hypoxia (Friedrich et al., 2014) resulting in increased OC preservation (Larowe et al., 2020). (c) The outer basin of Loch Etive is fed by Loch Awe a freshwater lake that acts as a natural sediment trap capturing the OC_terr_ before it reaches the lower basin. Loch Etive may be at the extreme end of the spectrum; but all multi‐basin fjords follow this pattern (high *F*_terr_ in the inner basin; lower *F*_terr_ in the outer basin). The most significant shifts in *F*_terr_ occur between fjord basins separated by shallow submarine sills (<20 m) such as Lochs Etive, Creran, Linnhe and Sunart (Figure S8 in Supporting Information S1), while the *F*_terr_ values fluctuate little in fjords with deep or no sills such as Lochs Melfort, Gairloch and Sullom Voe (Figure S7 in Supporting Information S1). These results indicate that the depth of these sills and the degree to which they restrict the exchange of water between basins is a mechanism that has a significant influence on the distribution of OC_terr_ and OC_mar_ across the fjord sediments.

**Figure 4:**
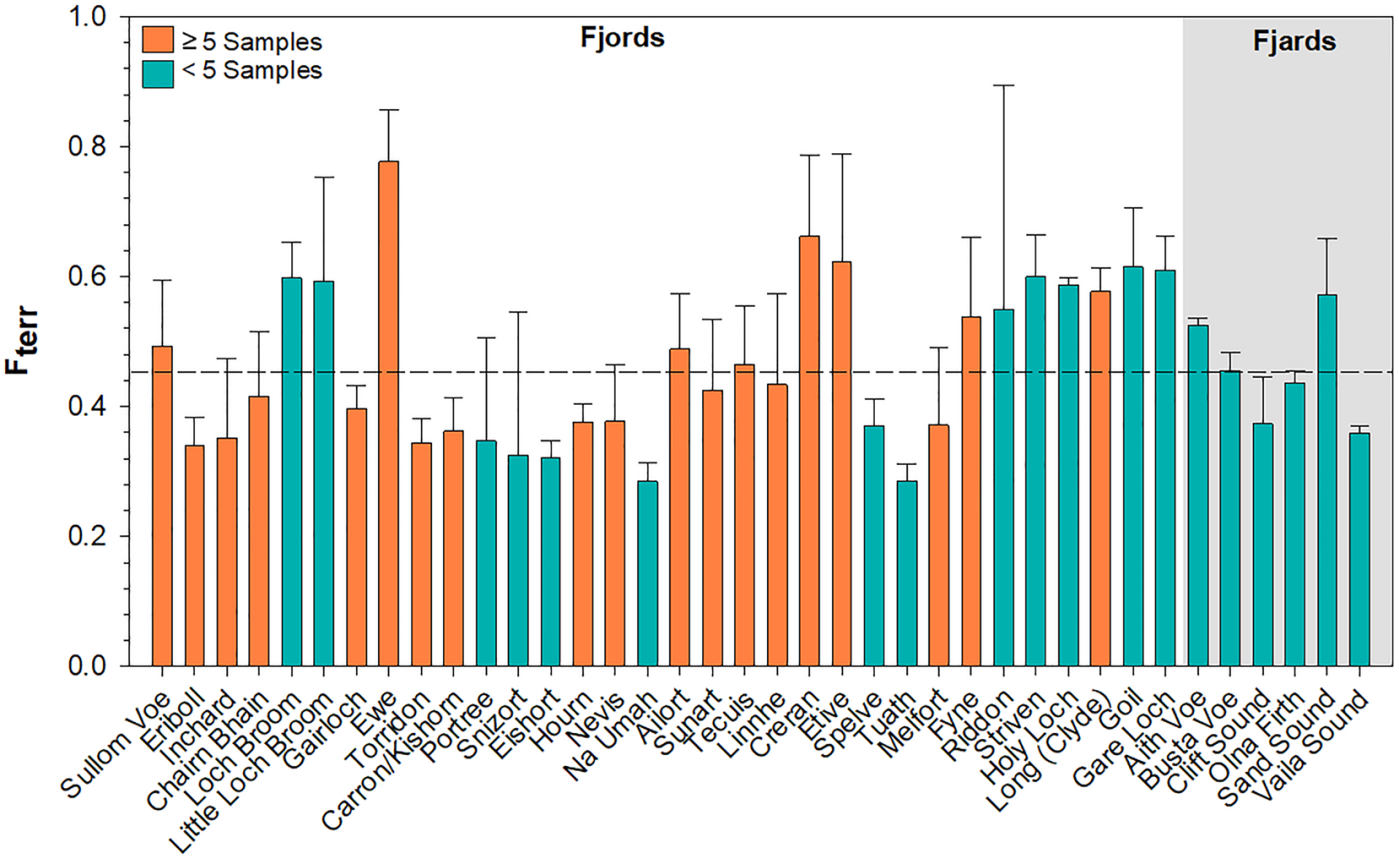
Estimated fraction of the organic carbon derived from the terrestrial environment and held within the surficial sediments of Scottish fjords. Dotted line represents the mean *F*_terr_ (0.47 ± 0.12) for all 38 systems studied.

Across the 38 fjords where we have data, on average 47% ± 12% of the OC within the surficial sediment originates from terrestrial sources (Figure 4). The highest whole fjord *F*_terr_ values are found in Loch Ewe (0.72 ± 0.08), Ailort (0.69 ± 0.09), Creran (0.66 ± 0.12), Teacuis (0.64 ± 0.09) and Etive (0.62 ± 0.17). These fjords all have shallow sills (Edwards & Sharples, 1986) and low oxygen bottom waters (i.e., Hypoxia) have been observed in Lochs Etive, Teacuis and Ailort (Friedrich et al., 2014; Gillibrand et al., 1996; Smeaton et al., 2016) likely driving the high *F*_terr_ values. The less restricted more geomorphologically open systems such as Loch Eriboll, Torridon, Melfort and Na Umah all have *F*_terr_ values <0.35 indicating that a greater proportion of the OC within these systems is derived from the marine environment.

### 4.3 A National Inventory of OC_terr_ in Scotland's Mid‐Latitude Fjords

Geomorphological, oceanographic and meteorological characteristics of the fjords (Edwards & Sharples, 1986) were compared to the calculated *F*_terr_ values from the 38 fjords (Figure 4) to determine if a relationship between these variables exist that could be exploited to allow the contribution of OC_terr_ to all Scottish fjords to be quantified. Initial examination of these characteristics indicated that smaller fjards were not comparable to the larger fjords. The disparity between these types of systems has previously been observed in Scotland, where the fjards of the Shetland Islands were categorized as distinct systems separate from the fjords of the mainland and Hebridian Islands (Lo Giudice Cappelli et al., 2019; Smeaton et al., 2017), therefore for the purposes of the study the six fjards (Figure 4) were excluded from the data mining exercise.

Of the multiple fjord characteristics tested (Table S2 in Supporting Information S1), outer sill depth (m) and tidal range (m) had the strongest linear correlations to *F*_terr_ (Table S3 and Figure S9 in Supporting Information S1). Both the outer sill depth and tidal range can be seen as proxies for the restrictiveness of the fjord which likely governs both the input of OC_mar_ and the retention of OC_terr_ within the fjord as conceptualized by Fuast and Knies (2019). When the outer sill depth and tidal range are compared with *F*_terr_ values there are reasonable correlations (Figure S10−S11 in Supporting Information S1). When combined the correlation between *F*_terr_ and these variables (outer sill depth (m) *x* tidal range (m)) is further improved (*R* ^2^ = 0.59; Figure S12 in Supporting Information S1). To determine if the quality of the regression model could be improved a partial least square (PLS) regression approach was applied to the data set (Table S2 in Supporting Information S1). PLS regression reduces the predictors to a smaller set of uncorrelated components and performs least squares regression (Tobias, 1995). The PLS regression again highlighted that the strong relationship between *F*_terr_, outer sill depth and tidal range exists, which is expressed as:

(4) $$F_{terr}=0.729-0.03845\times Tidal\;Range-0.003881\times Outer\;Sill\;Depth$$

**Figure 5:**
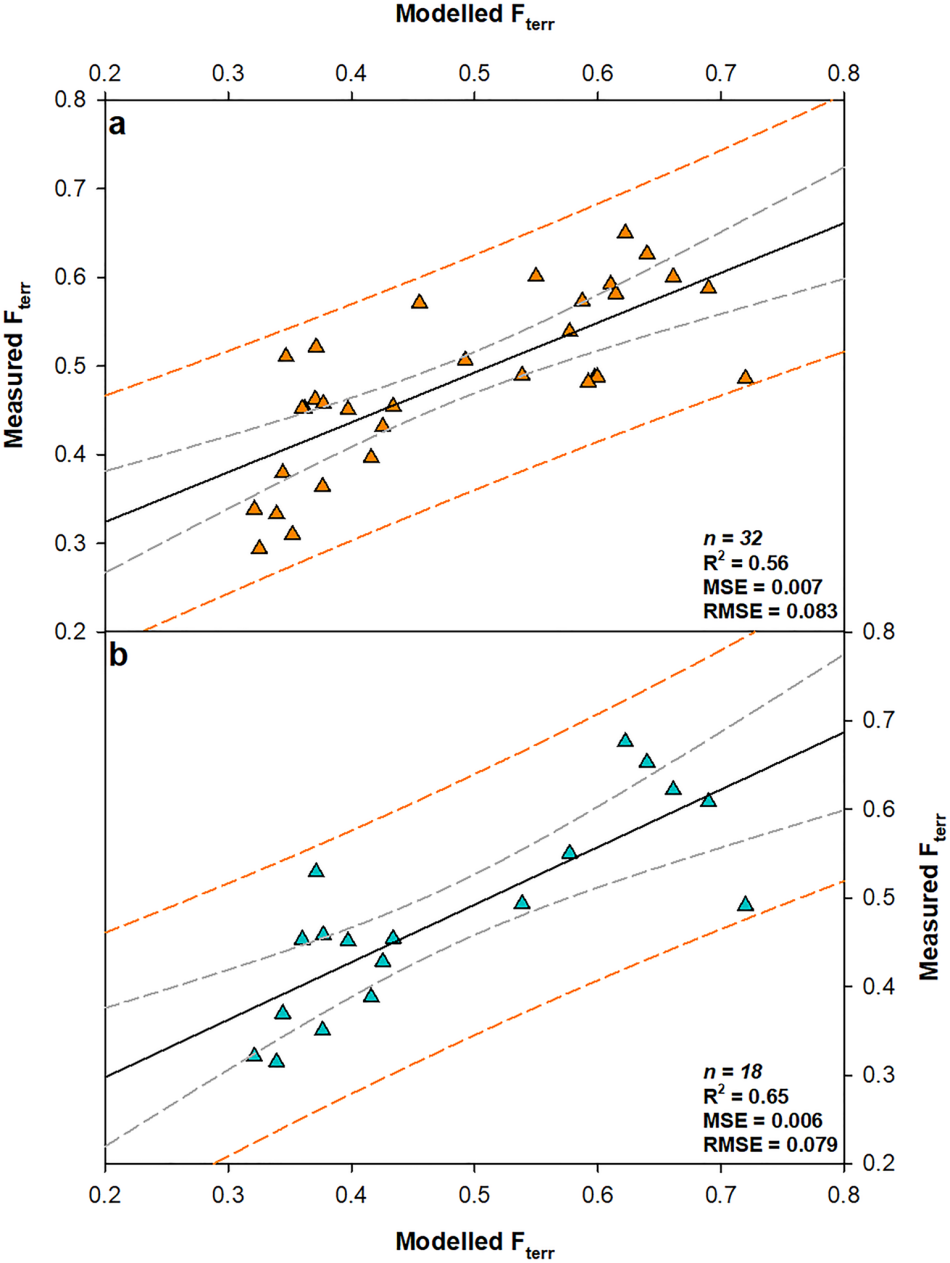
Comparison of modeled *F*_terr_ values produced using the partial least square (PLS) regression model to measured *F*_terr_ values. (a) All fjords (*n* = 32) where *F*_terr_ = 0.792–0.0401*Tidal Range–0.0054*Outer Sill Depth (b) Mainland fjords with ≥5 samples (*n* = 18). Dashed gray lines represent 95th percentile confidence intervals, dashed orange lines represent the upper and lower 95% prediction bands. Errors reported as mean‐squared error and root mean‐square error (RMSE). Additional outputs from the PSL regression can be found in Figure S13 in Supporting Information S1.

Utilizing the PLS approach, *F*_terr_ values were modeled using the tidal range and outer sill depth data (Figure 5). The modeled *F*_terr_ values produced from the PLS regression have a strong correlation with the measured values (*R* ^2^ = 0.56), the correlation is further improved when only the data from the mainland fjords with ≥5 samples are included (*R* ^2^ = 0.65). Additionally, the mean square error is reduced from 0.01 in the best performing liner regression (Figure S12 in Supporting Information S1) to 0.006 using the PLS regression model (Figure 5b). The comparison of these data sets provides confidence that the PLS regression model is an appropriate and statistically robust tool to estimate the OC_terr_ stored within all Scotland's fjord sediments.

Using the PLS regression model in conjunction with the tidal range and outer sill depth data compiled across all fjords (Edwards & Sharples, 1986) it is estimated that 52% ± 10% of the OC held within the sediments of Scottish fjords originates from the terrestrial environment (Figure S14 in Supporting Information S1). The outputs from the regression model highlight the Scottish fjords vary significantly from one another and fall into all four fjord categories outlined by Faust and Knies (2019) (*see* Section 3.4). The calculated *F*_terr_ values allow the Scottish fjords to be grouped (Figure S15 in Supporting Information S1) following the approach of Faust and Knies (2019) with 69% of systems falling into categories (a) or (b) (i.e., *F*_terr_ > *F*_mar_). The remaining fjords are split between categories (*c*) High oceanic water inflow and high freshwater inflow: Equal contributions of OC_mar_ and OC_terr_ (2%) and (d) High oceanic water inflow and low freshwater inflow: OC_mar_ dominated (29%) (Figure S15 in Supporting Information S1. Previous OC_terr_ estimates from Loch Sunart suggested that the majority of OC in the sediments is derived from the marine environment. Yet, this study estimates that OC_terr_ dominates (*F*_terr_ > 0.5) the vast majority (69%) of fjords.

**Figure 6:**
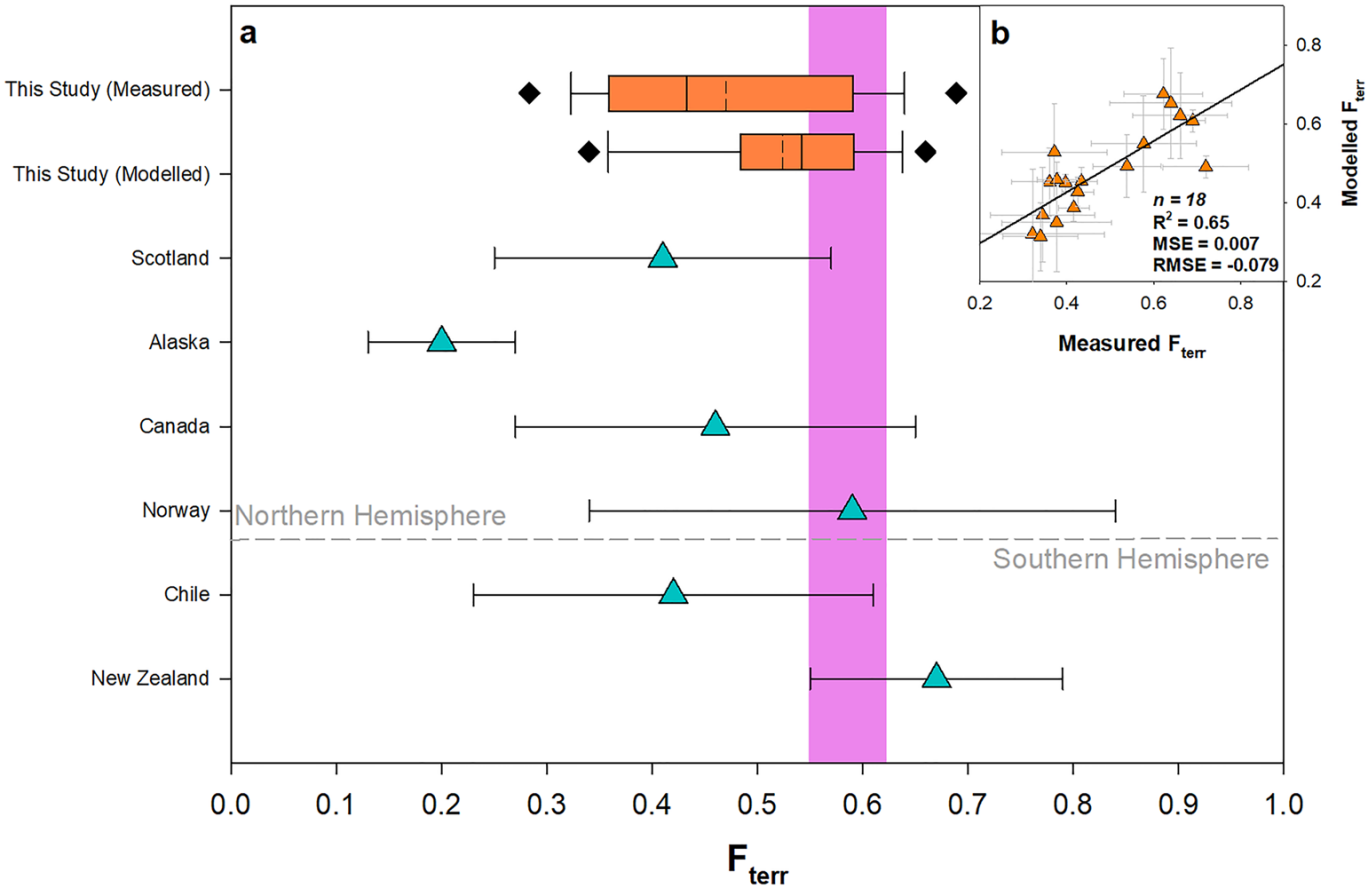
(a) Comparison of *F*_terr_ estimates from this study and other global systems (Bianchi et al., 2020; Cui, Bianchi, Savage, & Smith, 2016). Box plot—dotted line: mean; solid line: median; diamonds: 5th and 95th percentile. Purple shading represents the mean global *F*_terr_ value (Cui, Bianchi, Savage, & Smith, 2016). (b) Comparison of measured versus modeled *F*_terr_ values from the partial least square regression model utilizing data from the mainland fjords with ≥5 samples (*n* = 18). Mean‐square error and root mean square error represent the error associated with the regression model.

The quantity of OC_terr_ within the surficial sediments of Scottish fjords parallels the mean global estimates of between 55% and 62% (Cui, Bianchi, Savage, & Smith, 2016). When compared to individual regions (Figure 6) such as Patagonia (Chile) or Western Canada where it estimated that 42.3% and 47.5% of the OC is terrestrial in origin respectively (Bianchi et al., 2020; Cui, Bianchi, Savage, & Smith, 2016), the estimate from Scotland are higher potentially reflecting the carbon rich nature of the Scottish catchments (Lilly & Donnelly, 2012). Yet when compared to estimates from the fjords of New Zealand (OC_terr_: 73.4%) and East Canada (OC_terr_: 70.1%) the values are meaningfully lower, highlighting a clear difference between these systems that has potentially been overlooked in the compilation of global estimates (Figure 6).

Unlike the Scottish fjords (McIntyre & Howe, 2010; Syvitski & Shaw, 1995) the systems in New Zealand are highly restricted due to their geomorphology which leads to hypoxic and in some places anoxic bottom waters (Cui, Bianchi, Savage, & Smith, 2016; Hinojosa et al., 2014; Ramirez et al., 2016; Smith et al., 2010). These factors lead to the burial and preservation of large quantities of OC_terr_. Until recently, there have been few studies investigating the contribution of OC_terr_ to fjord sediments in geomorphologically less restricted and open systems. Recent work in Norway suggests that the more open fjords are heavily influenced by coastal currents (e.g., NAC) which drive OC_mar_ into these relatively well‐exchanged fjords (Faust & Knies, 2019). Contrary to what is observed in New Zealand where the high OC burial rates are driven by OC_terr_ input (Cui, Bianchi, Jaeger, & Smith, 2016; Ramirez et al., 2016; Smith et al., 2015), the increased OC_mar_ input observed in Norwegian and potentially Scottish fjords results in above average OC burial rates (Faust & Knies, 2019; Smeaton, Hunt, et al., 2021). The data from this study partially supports the findings of these studies; that in fjords with muted submarine geomorphology (i.e., deep or no sills) local oceanographic currents such as the SCC (Figure 1) facilitate the input of OC_mar_ into the fjords. In these systems (29% Scottish fjords) the OC_terr_ contribution is lower than current global estimates (Cui, Bianchi, Savage, & Smith, 2016), yet the sediment stores an equivalent and in some cases greater quantity of OC than those dominated by OC_terr_ (Table S6 in Supporting Information S1).

**Figure 7:**
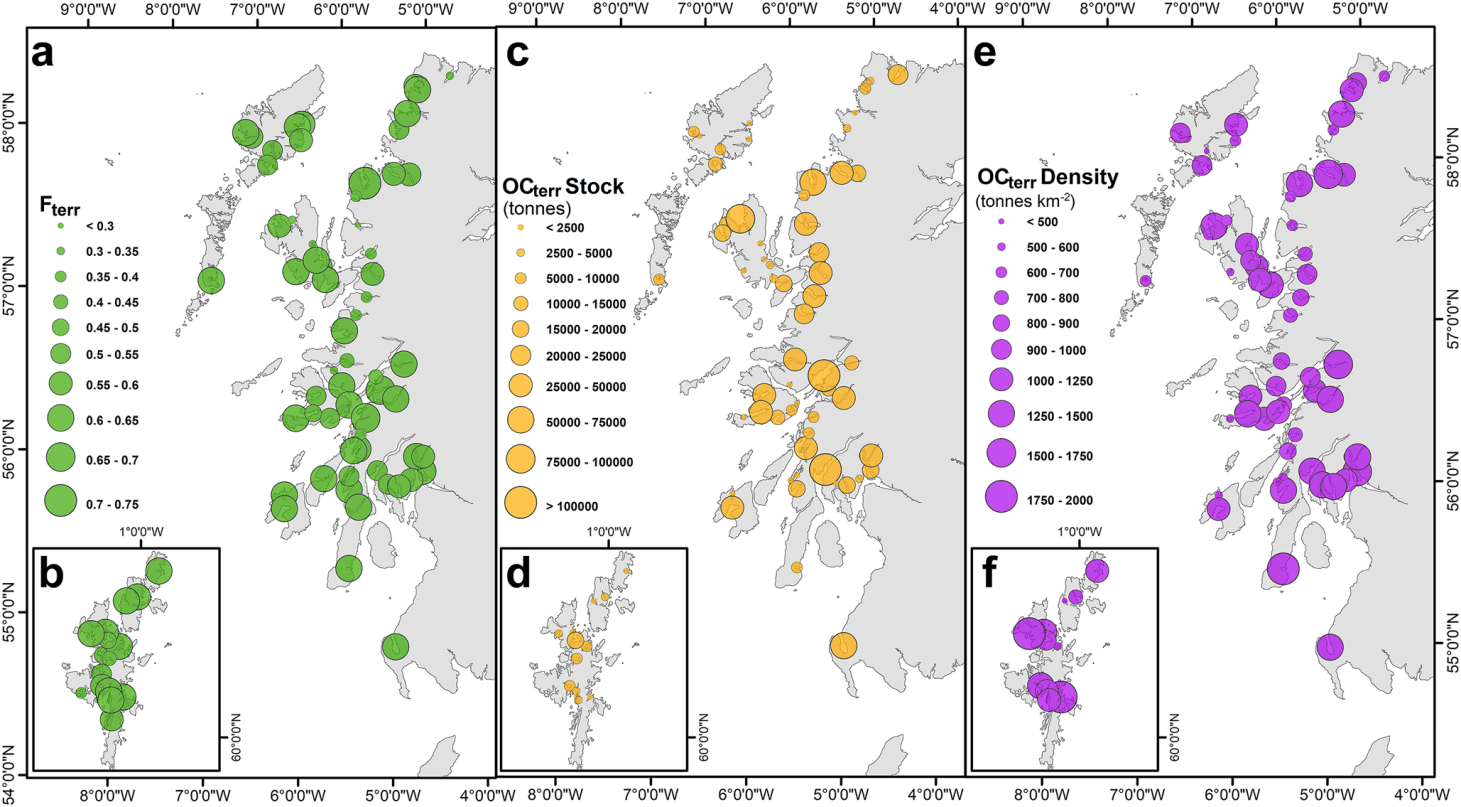
Surficial sediment (top 10 cm) OC_terr_ (a and b) *F*_terr_ values. (c andd) OC_terr_ stock (tonnes OC) (e and f) OC_terr_ densities (tonnes OC km^−2^) from across the mid‐latitude fjords of Scotland. Further breakdown of the data can be found in Tables S4 and S5 in Supporting Information S1.

By combining the *F*_terr_ values from each of the fjords (Figure 7a) with surficial sediment (top 10 cm) OC stocks (Smeaton & Austin, 2019; Smeaton, Yang, & Austin, 2021) it is estimated that there is 1.54 ± 0.29 Mt OC_terr_ held within the surface sediments of Scotland's fjords and a further 0.06 ± 0.01 Mt OC_terr_ held with the fjards (Figures 7c‐7d). The surficial (top 10 cm) sediment OC_terr_ stocks range between 324 ± 17 tonnes OC_terr_ in Loch Don (Island of Mull) and 246,457 ± 55,586 tonnes OC_terr_ in Loch Fyne (Figure 7b). The greatest OC_terr_ stocks are located within the largest fjords, yet the surficial sediments of smaller more restricted fjords contain more OC_terr_ when normalized for area (Figure 7c). The fjords with the highest OC_terr_ densities tend to be smaller single basin systems with shallow outer sills (<8 m) such as Loch Craignish (2,181 ± 80 tonnes OC_terr_ km^−2^) and Loch Feochan (1,986 ± 101 tonnes OC_terr_ km^−2^). The OC densities in these systems far exceed those observed in multi‐basin systems such as Loch Etive (1,344 ± 358 tonnes OC_terr_ km^−2^) and Loch Creran (1,122 ± 214 tonnes km^−2^) where a greater mix of OC derived from the terrestrial and marine environments are found in the sediments.

### 4.4 Terrestrial OC Loss Across the Land‐Ocean Interface

**Figure 8:**
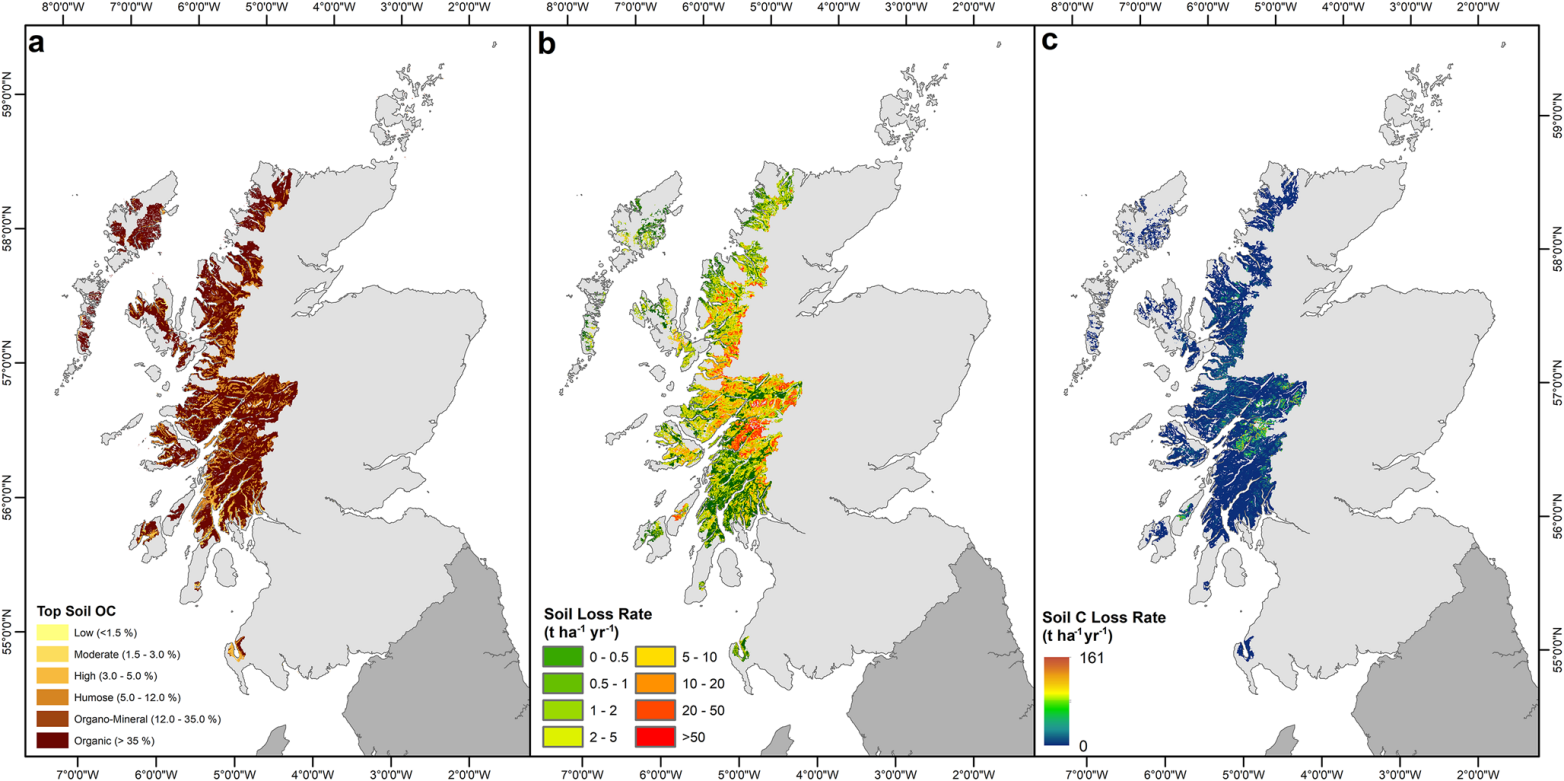
Erosion and transport of soil organic carbon (OC) by water across the catchments of the mid‐latitude fjords of Scotland. (a) Top soil (top 15 cm) OC content (Lilly & Donnelly, 2012); (b) Soil loss rate based on the Revised Universal Soil Loss Equation model (Panagos et al., 2015); (c) Estimated soil OC loss rate from across the fjord catchments.

The catchments of Scotland's fjords cover an area of 14,424 km^2^. Within these catchments, some of the most OC rich soils within the UK are found, with soil OC values commonly >35% (Figure 8a). A significant area of these soils are also highly vulnerable to erosion by water (Panagos et al., 2015) with soil loss rates ranging between 0.5 and >50 tonnes ha^−1^ yr^−1^ (Figure 8b). Annually, it is estimated that within these catchments between 4.34 and 5.78 (mean: 5.06) Mt OC is mobilized by water (Figure 8c). The majority (∼90%) of the mobilized OC will be redeposited within the individual catchments (Meade et al., 1990; Stallard, 1998) with between 0.43 and 0.58 (mean: 0.51) Mt of OC reaching the fjords.

## 5 First Order Sedimentary OC Budget for Scotland's Fjords

**Figure 9:**
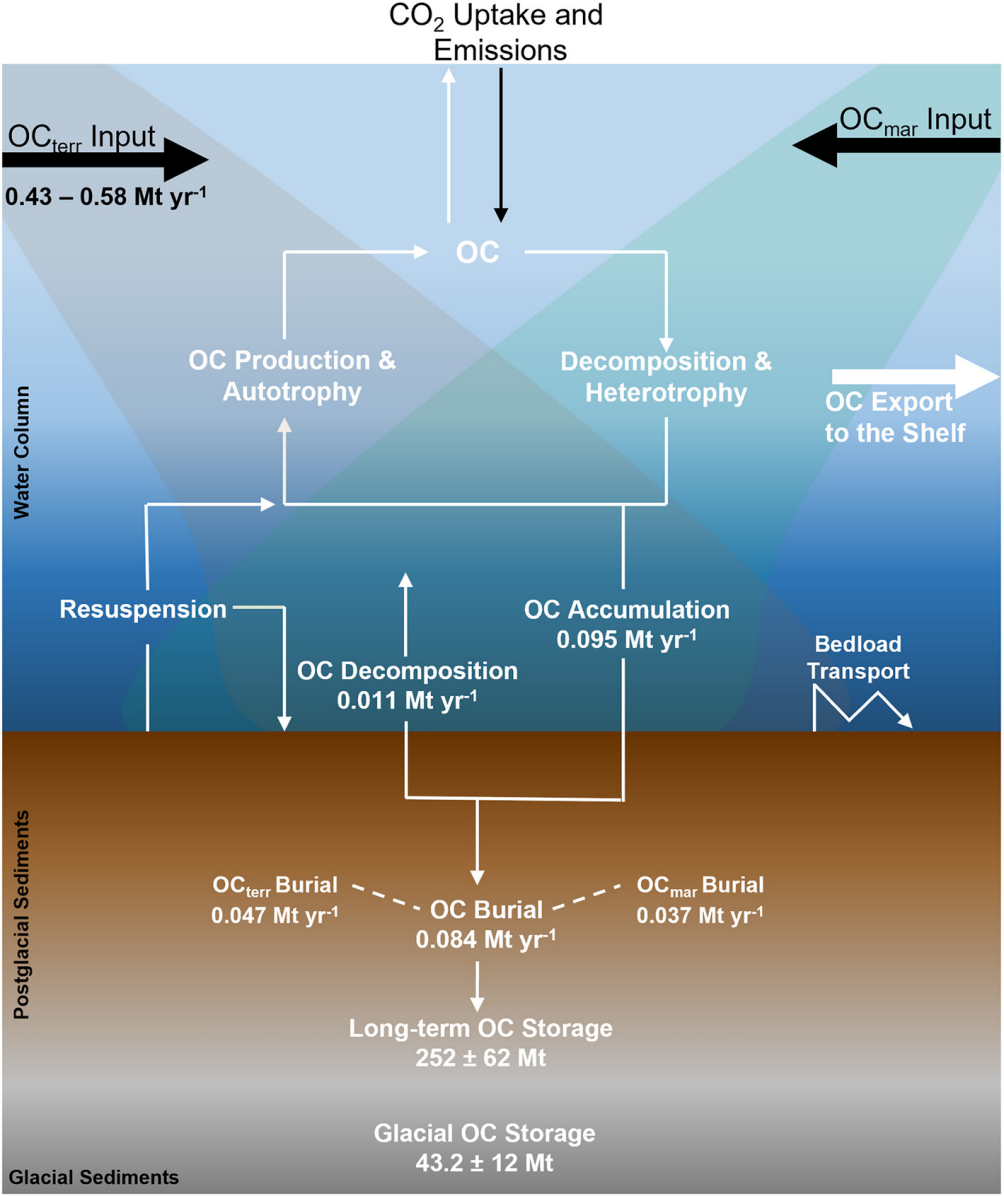
First‐order organic carbon (OC) budget for the mid‐latitude fjords of Scotland. Black Arrows: External sources. White Arrows: highlight the pathways through which the OC input from external sources are processed within the water column and sediment of the fjords.

By integrating our improved understanding of OC_terr_ dynamics across the land‐ocean interface with existing surficial (Smeaton & Austin, 2019; Smeaton, Yang, & Austin, 2021) and depth integrated (Smeaton et al., 2016, 2017) sedimentary OC stocks and OC accumulation and burial rates (Smeaton, Hunt, et al., 2021) it is possible to create a first‐order sedimentary OC budget for the mid‐latitude fjords of Scotland (Figure 9).

The surficial (top 10 cm) sediments within Scotland's fjords hold an estimated 3.9 ± 0.6 Mt of OC (Smeaton, Yang, & Austin, 2021). By combing the fjord specific *F*_terr_ values (Figure S14 in Supporting Information S1) and surficial (top 10 cm) sediment OC stocks (Smeaton & Austin, 2019) it is estimated that 1.54 ± 0.29 Mt of the OC within the surficial sediments of Scotland's fjords originates from the terrestrial environment. Annually, a further 95,000 tonnes of OC accumulates on the seabed with 84,000 tonnes of OC (Smeaton, Hunt, et al., 2021) being integrated into the existing 252 ± 62 Mt OC stored within the post‐glacial sediments (Smeaton et al., 2017) (Figure 9). The processes which govern the quantity of this newly deposited OC as it becomes part of the larger sedimentary OC store differ between fjords and even different depositional settings within a single fjord (Arndt et al., 2013; Bianchi et al., 2020; Middelburg, 2018). In general, the effectiveness of marine sediments at retaining OC is driven by bottom water oxygen concentrations, bioturbation, temperature, transport time from source to deposition, sediment geochemistry and natural or anthropogenic disturbance (Arndt et al., 2013; Larowe et al., 2020; Middelburg, 2018). The sediments within Scottish fjords are highly effective at storing OC, with a burial efficiency of 79% ± 16%, which results in 84,000 tonnes OC being buried annually and a 11,000 tonnes lost through sedimentological and remineralization processes (Smeaton, Hunt, et al., 2021) (Figure 9).

Each year it is estimated that between 0.43 and 0.58 Mt OC enters the fjords of Scotland from the terrestrial environment (Figure 8). Annually, 84,000 tonnes of OC is buried in Scottish fjord sediments (Smeaton, Hunt, et al., 2021) using the mean OC_terr_ value (52% ± 10%) this equates to 0.044 ± 0.008 Mt OC_terr_ yr^−1^ being buried in these sediments which represents between 7.6% and 10.2% of the OC lost from the adjacent catchments. Generally, within terrestrial C budgets, the OC entering the coastal ocean would be considered to have been remineralized and lost to the atmosphere as CO_2_, when in reality coastal and inshore sediments capture and lock away a portion of this “lost” OC. Within the sediments of Scotland's fjords 160,306 ± 30,828 tonnes CO_2_eq originating from the terrestrial environment is locked away each year, however this trapped and stored OC is currently considered to have been lost and remineralized to the atmosphere and is therefore not or mis‐accounted for within current terrestrial OC budgets.

## 6 Conclusion

Terrestrially derived OC is a core component of the sediments stored within the mid‐latitude fjords of Scotland, with 52% ± 10% of the OC held within the surficial sediments (top 10 cm) originating from the terrestrial environment. Though less common the sediments within 29% of Scottish fjords store significant quantities (OC_mar_ ≥ 0.5) of OC derived from the marine environment, while less common these marine dominated systems play and equivalent role in the storage of OC and long‐term climate regulation as their OC_terr_ dominated counterparts.

Across Scottish fjord sediments, the terrestrial environment is the dominant source of OC with an estimated 1.54 ± 0.29 Mt OC_terr_ being held within their surficial sediments with 0.044 ± 0.008 Mt OC_terr_ yr^−1^ being buried. Annually, this equates to between 7.6% and 10.2% of the terrestrial OC loss from fjord catchments. Many existing terrestrial OC budgets treat the OC lost from the terrestrial environment through rivers to the marine environment as a loss of CO_2_ to the atmosphere. Our findings highlight that the coastal ocean and fjords in particular, provide a currently unrecognized climate service by capturing and delivering effective long‐term storage for a portion of this “lost” OC_terr_ that needs to be better accounted for within national and global carbon budgets.

## Conflict of Interest

The authors declare no conflicts of interest relevant to this study.

## Supporting information

Supporting Information S1

Data Set S1

## Data Availability

All data produced as part of this study can be found in the Supporting Information S1.
