# Supplementary material for: Understanding the Role of Terrestrial and Marine Carbon in the Mid‐Latitude Fjords of Scotland
Source: Global Biogeochem Cycles. 2022 Nov 11;36(11):e2022GB007434. doi: 10.1029/2022GB007434 (PMC9786263; doi:10.1029/2022GB007434)
Supplement: Supplementary file 1 — Supporting Information S1 [file GBC-36-e2022GB007434-s002.docx]

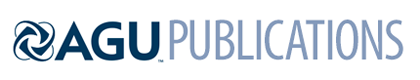


*Global Biogeochemical Cycles*

Supporting Information for

**Understanding the Role of Terrestrial and Marine Carbon in the Mid-Latitude Fjords of Scotland.**

C. Smeaton^1*^ and W.E.N. Austin^1,2^

^1^School of Geography & Sustainable Development, University of St Andrews, St Andrews, KY16 9AL, UK.

^2^Scottish Association for Marine Science, Oban, PA37 1QA, UK.

*Corresponding Author: Craig Smeaton ([cs244@st-andrews.ac.uk](mailto:cs244@st-andrews.ac.uk))

**Contents of this file**

**Supplementary Figure 1.** Hydrological basins that drain to Scottish fjords.

**Supplementary Figure 2.** Sampling locations from seven fjords on the North West coast of Scotland.

**Supplementary Figure 3.** Sampling locations from eight fjords on the West coast of Scotland.

**Supplementary Figure 4.** Sampling locations from six fjords on the South West coast of Scotland.

**Supplementary Figure 5.** Sampling locations from six fjards across the Shetland Islands.

**Supplementary Figure 6.** Frequency plots of geochemical measurements from 450 sediment samples

**Supplementary Figure 7.** F_terr_ values from the head to the mouth of sixteen Scottish fjords.

**Supplementary Figure 8.** F_terr_ held with the sediments of different fjord basins.

**Supplementary Figure 9.** Correlation matrix for the fjord variables.

**Supplementary Figure 10.** The relationship between F_terr_ and the depth of the outer sill.

**Supplementary Figure 11**. The relationship between F_terr_ and tidal range.

**Supplementary Figure 12.** The relationship between F_terr_ and tidal range x outer sill depth.

**Supplementary Figure 13.** Partial least square regression outputs.

**Supplementary Figure 14**. Modelled F_terr_ estimates for the mid-latitude fjords of Scotland.

**Supplementary Figure 15.** Fjords categorized following the Faust and Knies, (2019) scheme.

**Supplementary Table 1.** Mean F_terr_ of the surficial sediments in each of the fjord basins in this study.

**Supplementary Table 2.** Summary statistics for the environmental variables from the 32 fjords. **Supplementary Table 3.** Correlation and covariance tests between F_terr_ and fjord variables.

**Supplementary Table 4.** Mainland fjords ranked by F_terr,_ OC_terr_ stock and OC_terr_ density.

**Supplementary Table 5.** Fjards ranked by F_terr_, OC_terr_ stock and OC_terr_ density.

**Supplementary Table 6.** Scottish fjords OC Storage grouped using the Faust and Knies, (2019) scheme.

**Additional Supporting Information (Files uploaded separately)**

**Datasets S1:** Dataset (*Excel*)


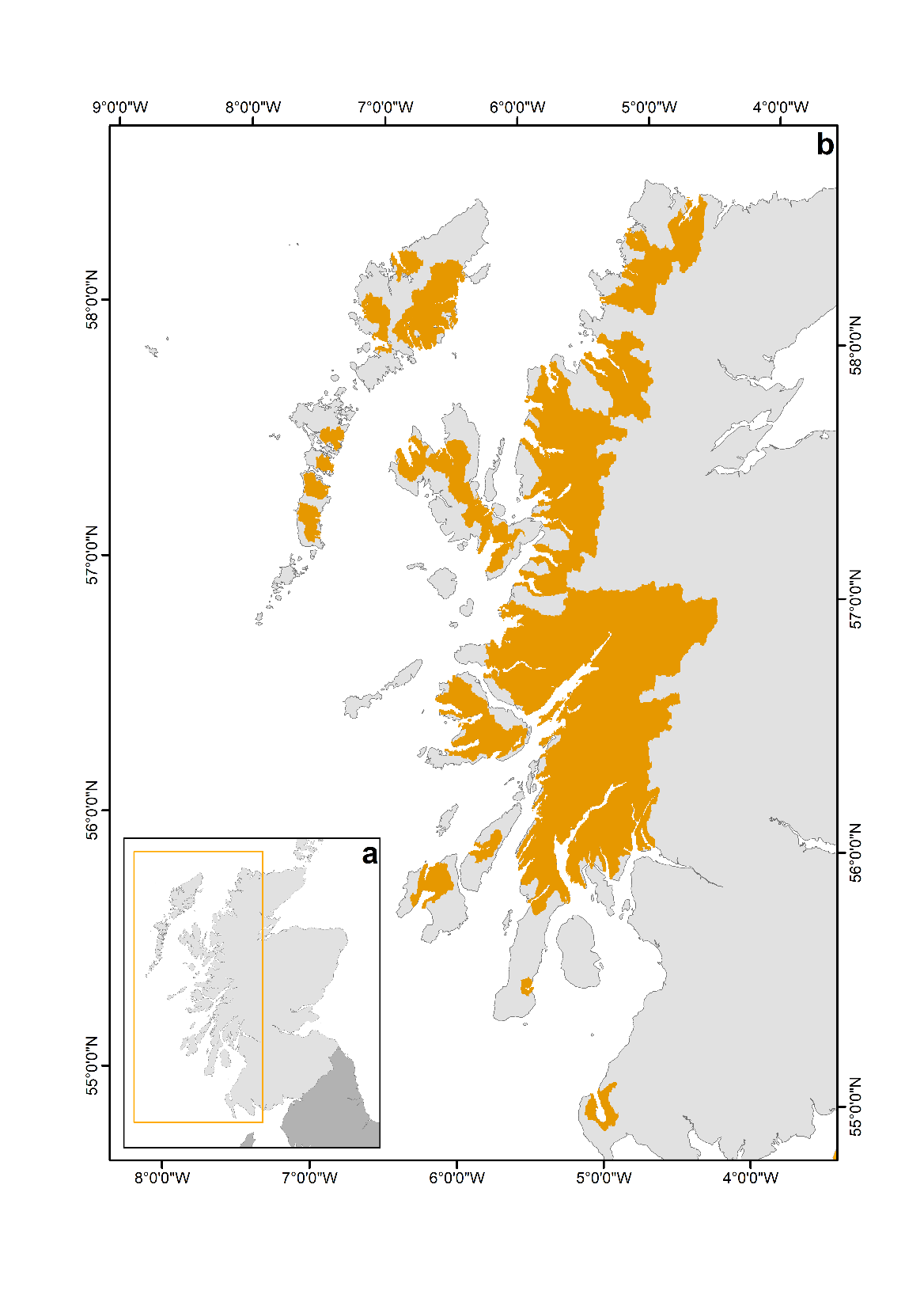


**Supplementary Figure 1.** Hydrological basins that drain to Scottish fjords.


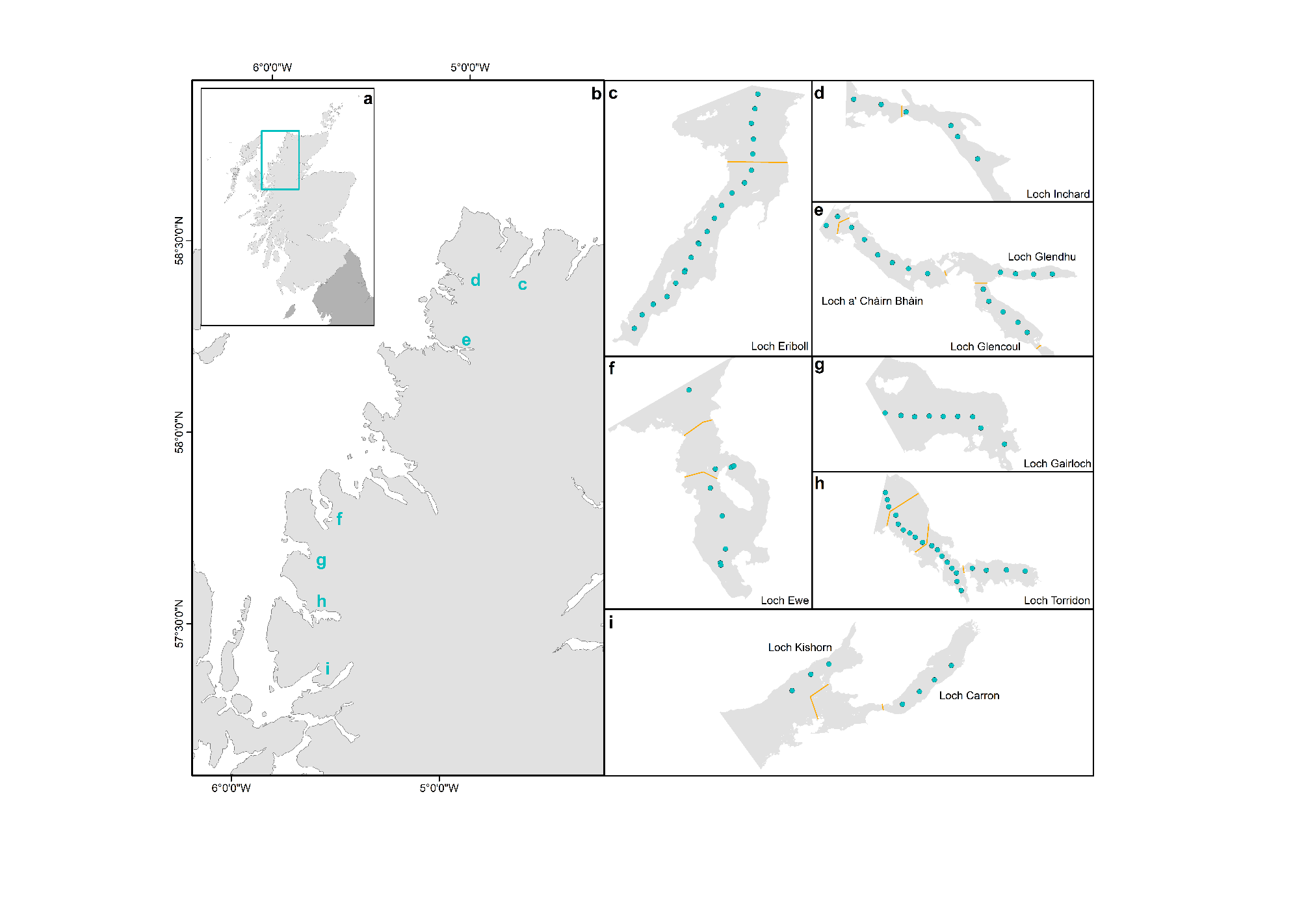


Supplementary Figure 2. Sampling locations from seven fjords on the northwest coast of Scotland. Orange lines highlight the locations of the submarine sills (Edwards and Sharples, 1986).


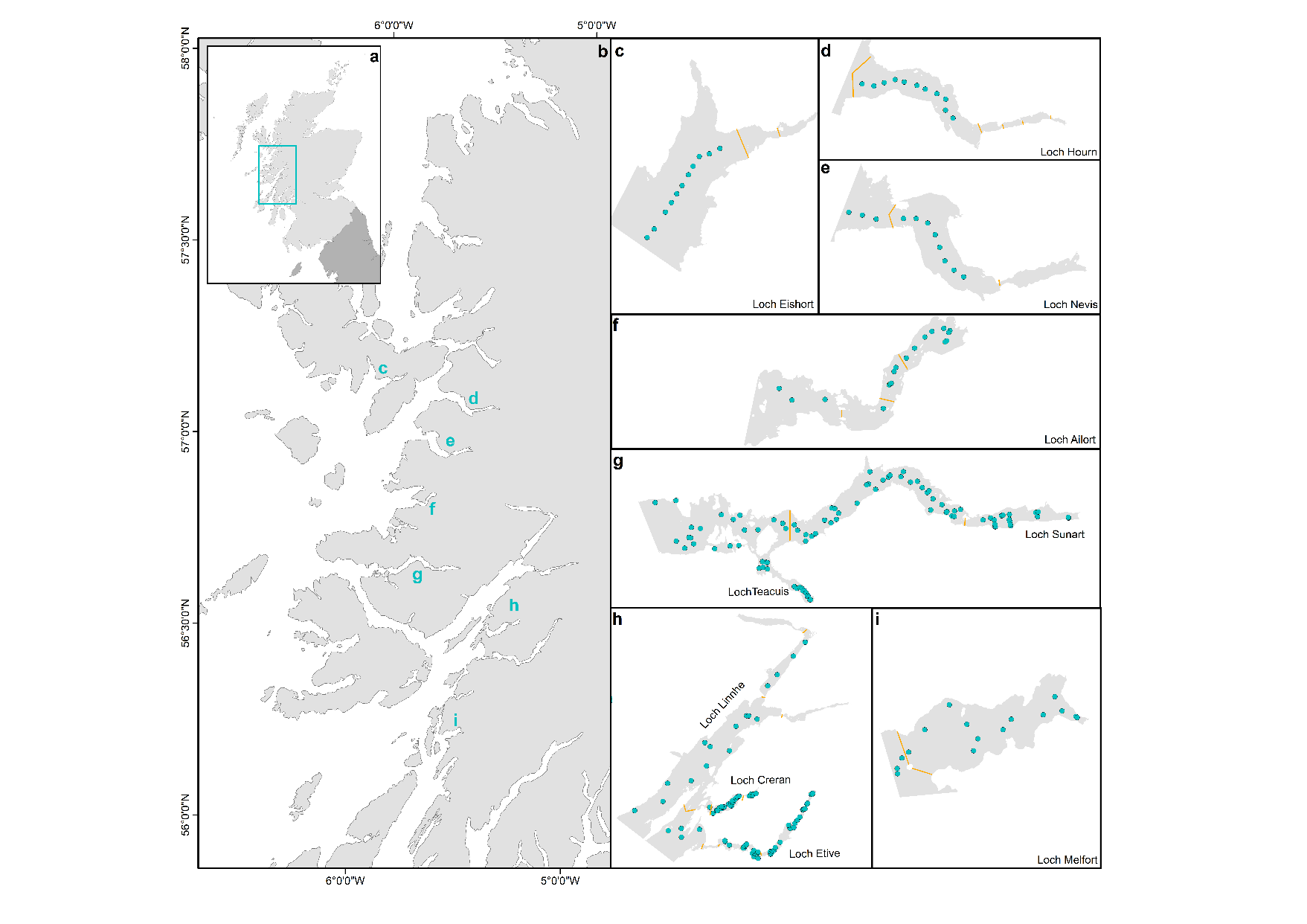


Supplementary Figure 3. Sampling locations from eight fjords on the west coast of Scotland. Orange lines highlight the locations of the submarine sills (Edwards and Sharples, 1986).


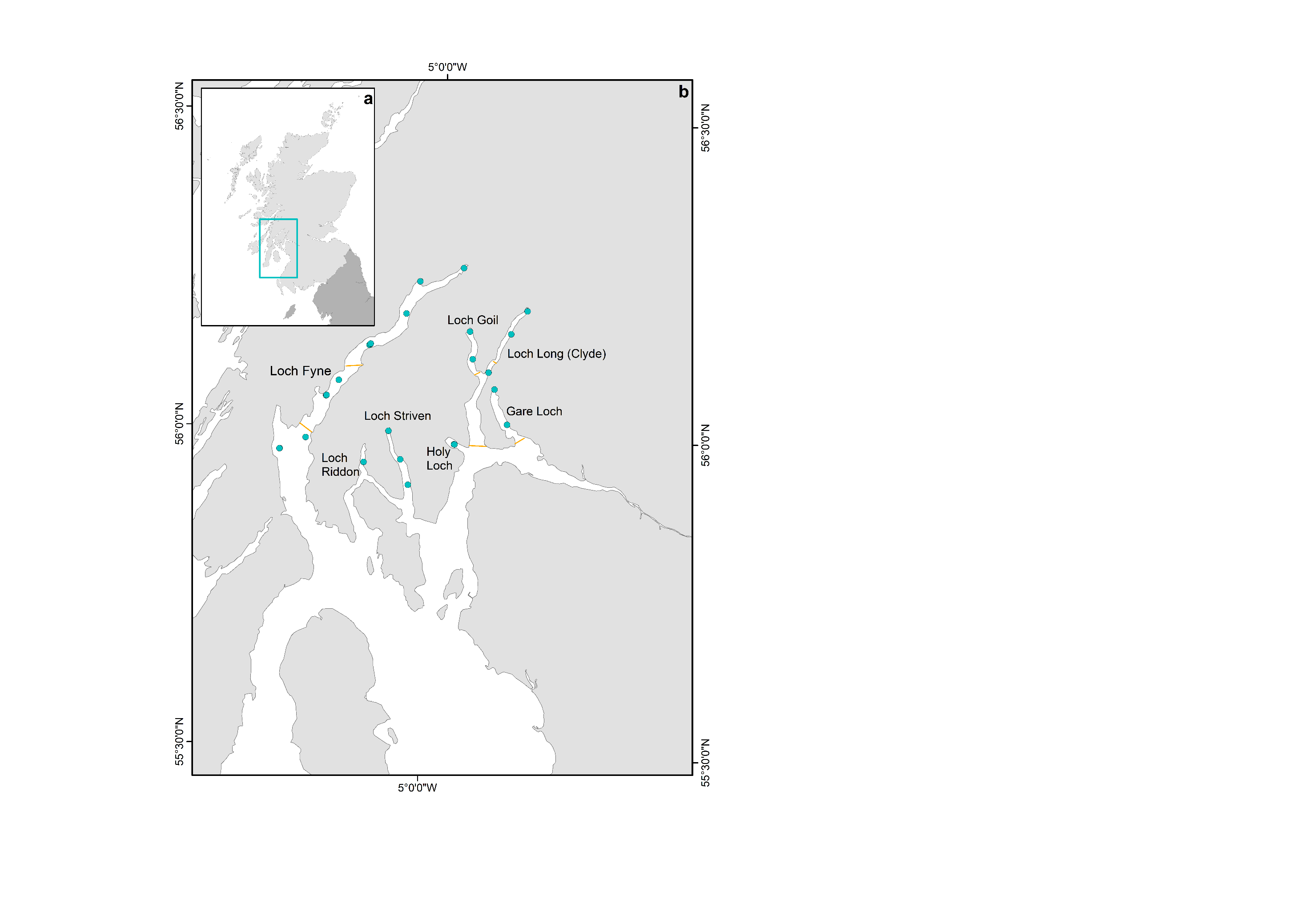


Supplementary Figure 4. Sampling locations from six fjords on the southwest coast of Scotland. Orange lines highlight the locations of the submarine sills (Edwards and Sharples, 1986).


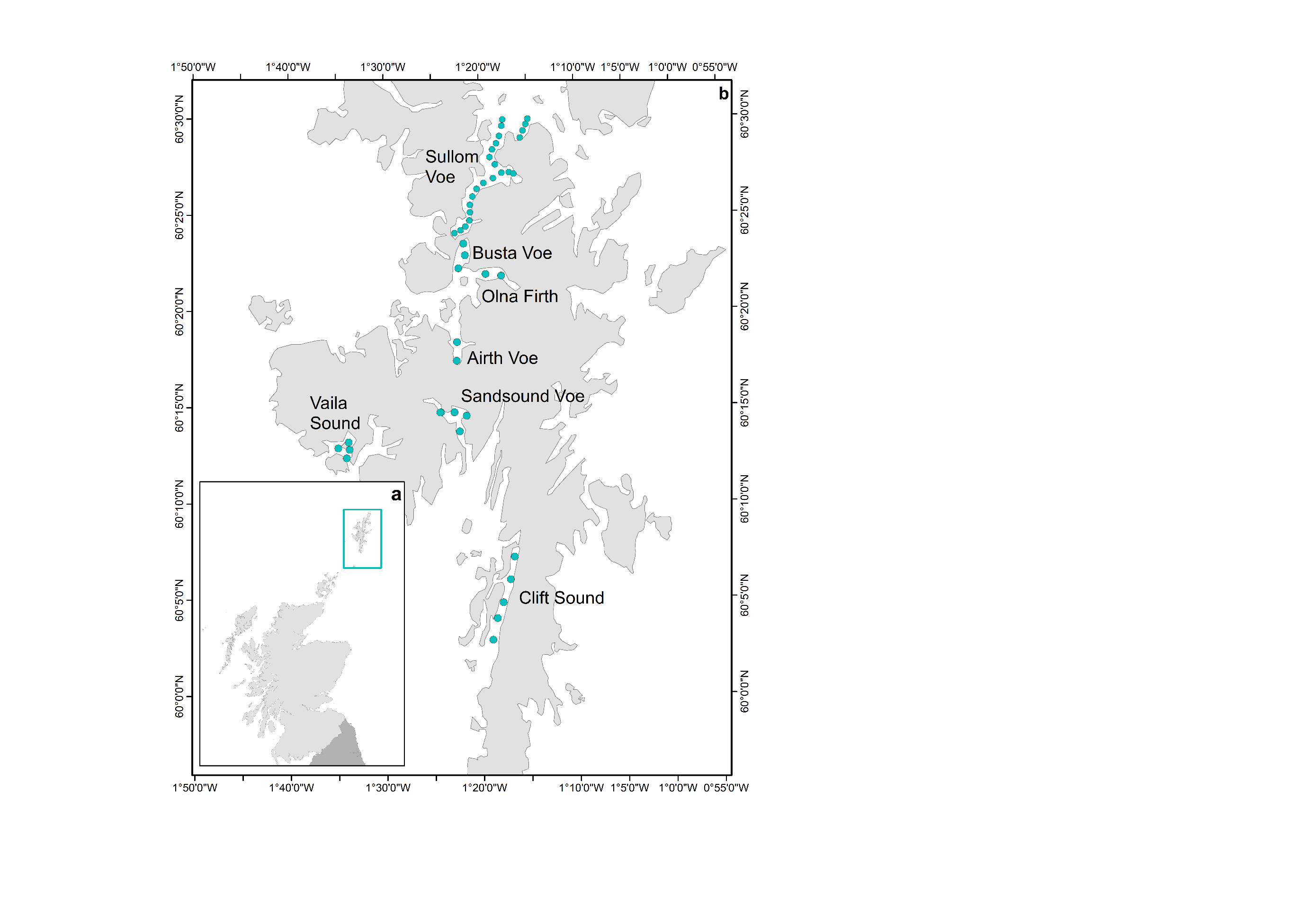


Supplementary Figure 5. Sampling locations from Sullom Voe and six fjards across the Shetland Islands.


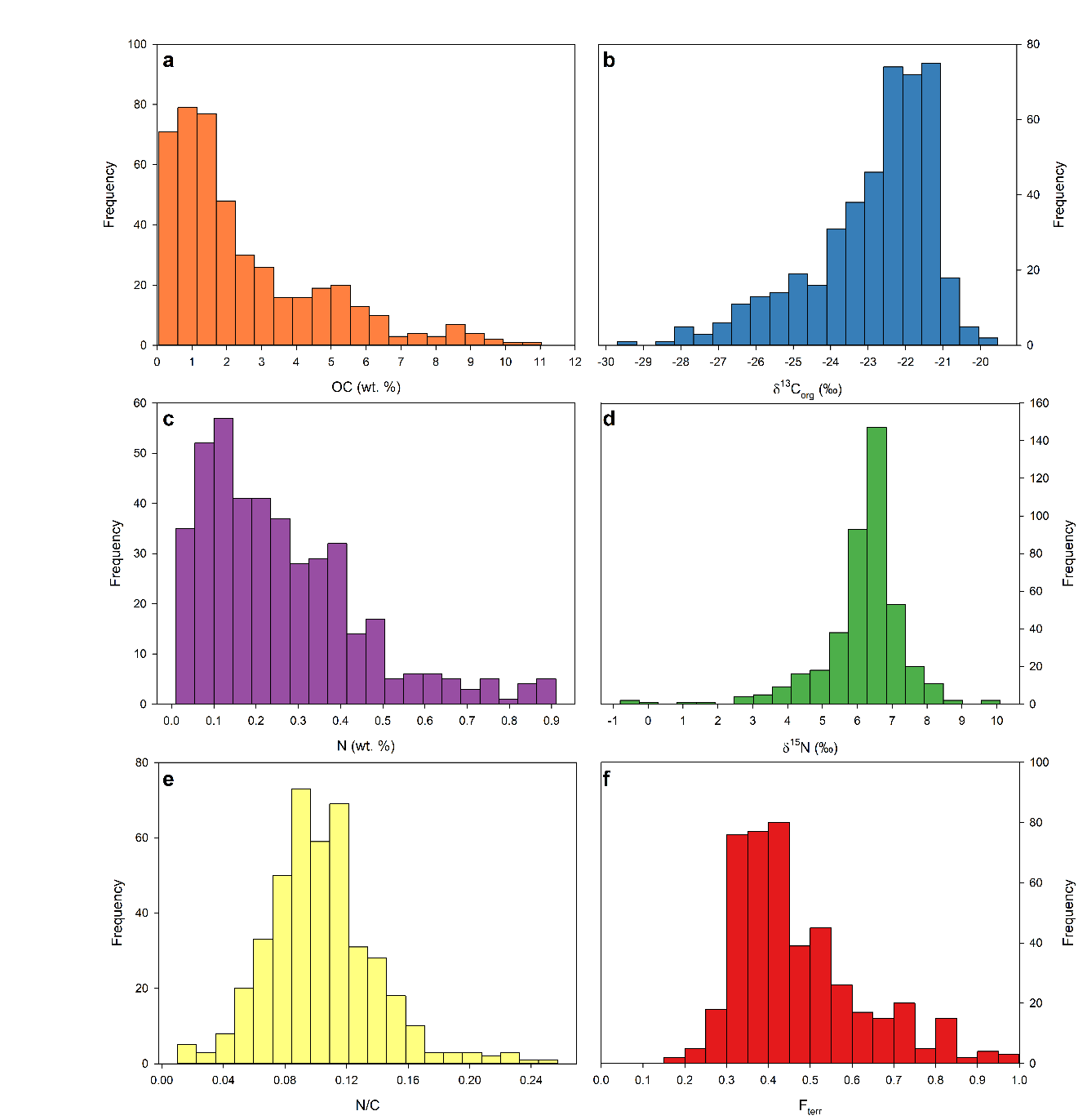


Supplementary Figure 6. Frequency plots of geochemical measurements for the 450 surface samples (a) OC (wt. %), (b) ẟ^13^C_org_ (‰), (c) N (Wt. %), (d) ẟ^15^N (‰), (e) N/C (*molar*) and (f) F_terr_.


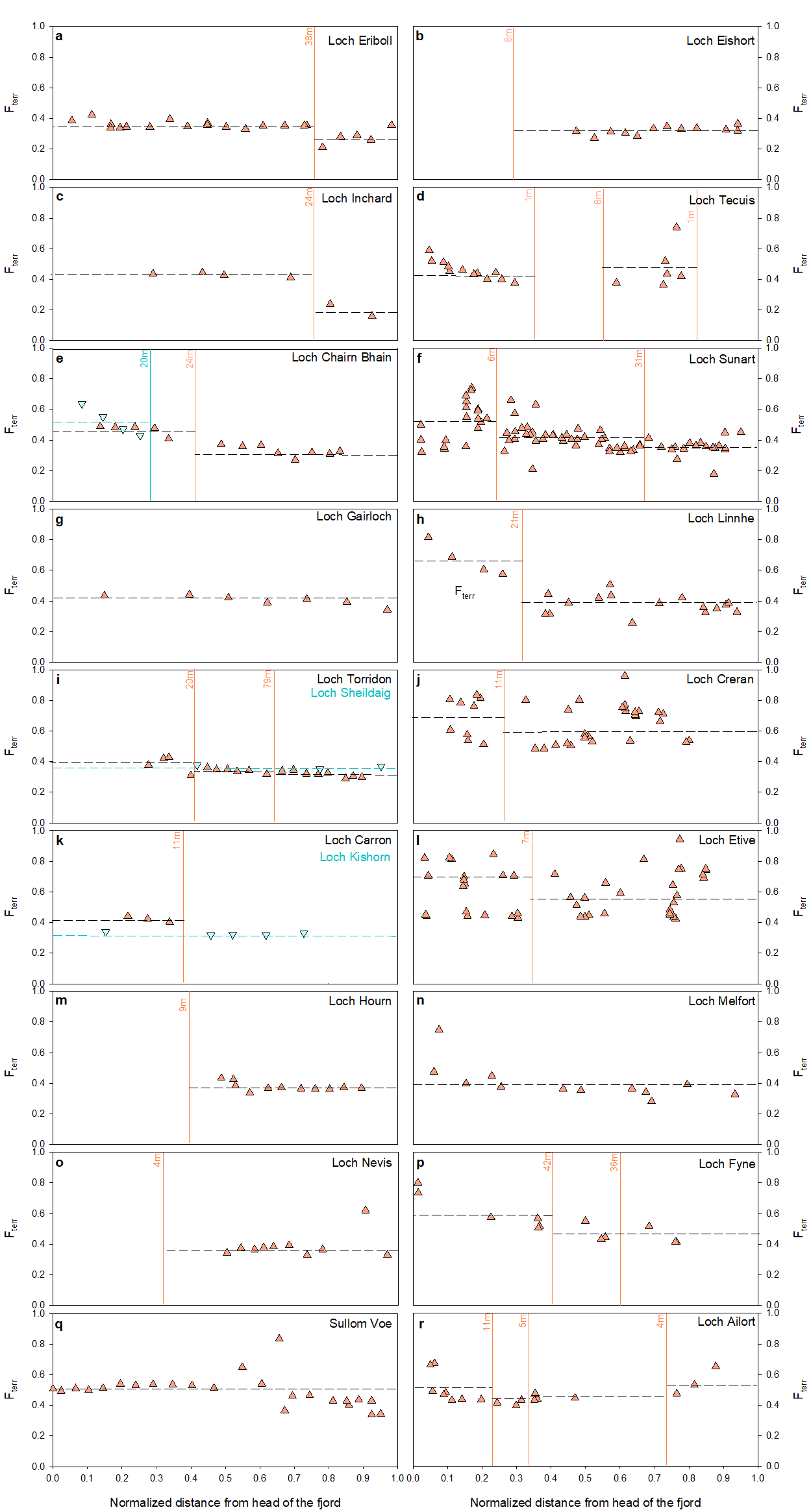


**Supplementary Figure 7.**  F_terr_ values from the head to the mouth of sixteen Scottish fjords with the greatest number of samples. Vertical lines indicate the location and water depth of the submarine sills. Dotted lines represent the mean F_terr_ value for each basin. All normalized using the total head to outer sill distance.


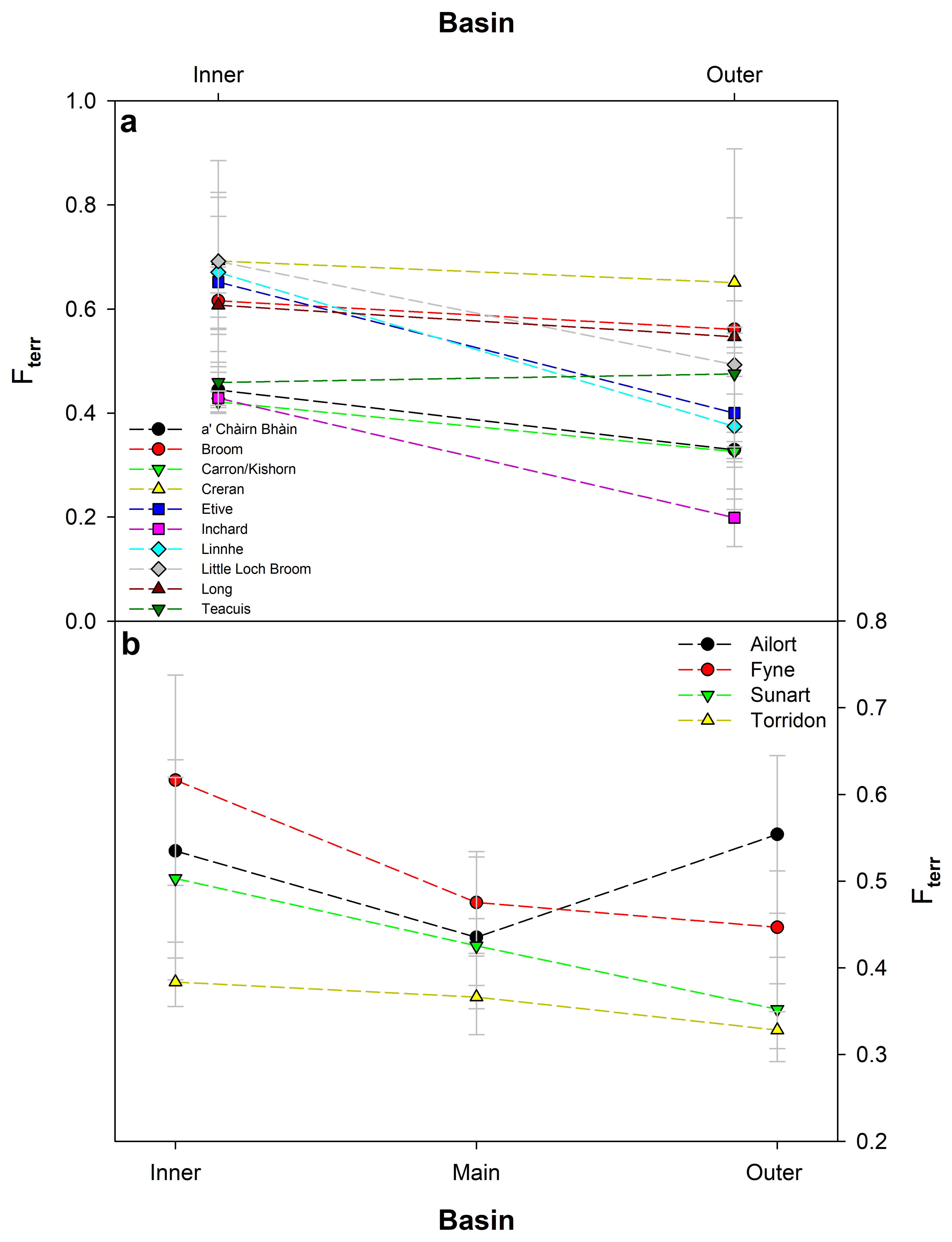


**Supplementary Figure 8.** Fraction of the OC that originates from the terrestrial environments held within the surficial sediments of the different basins of (**a**) double basin and (**b**) triple basin Scottish fjords.

**Supplementary Figure 9.** Correlation matrix (*r value*) for the fjord variables from across 32 systems (Data summarized in Table. S3).


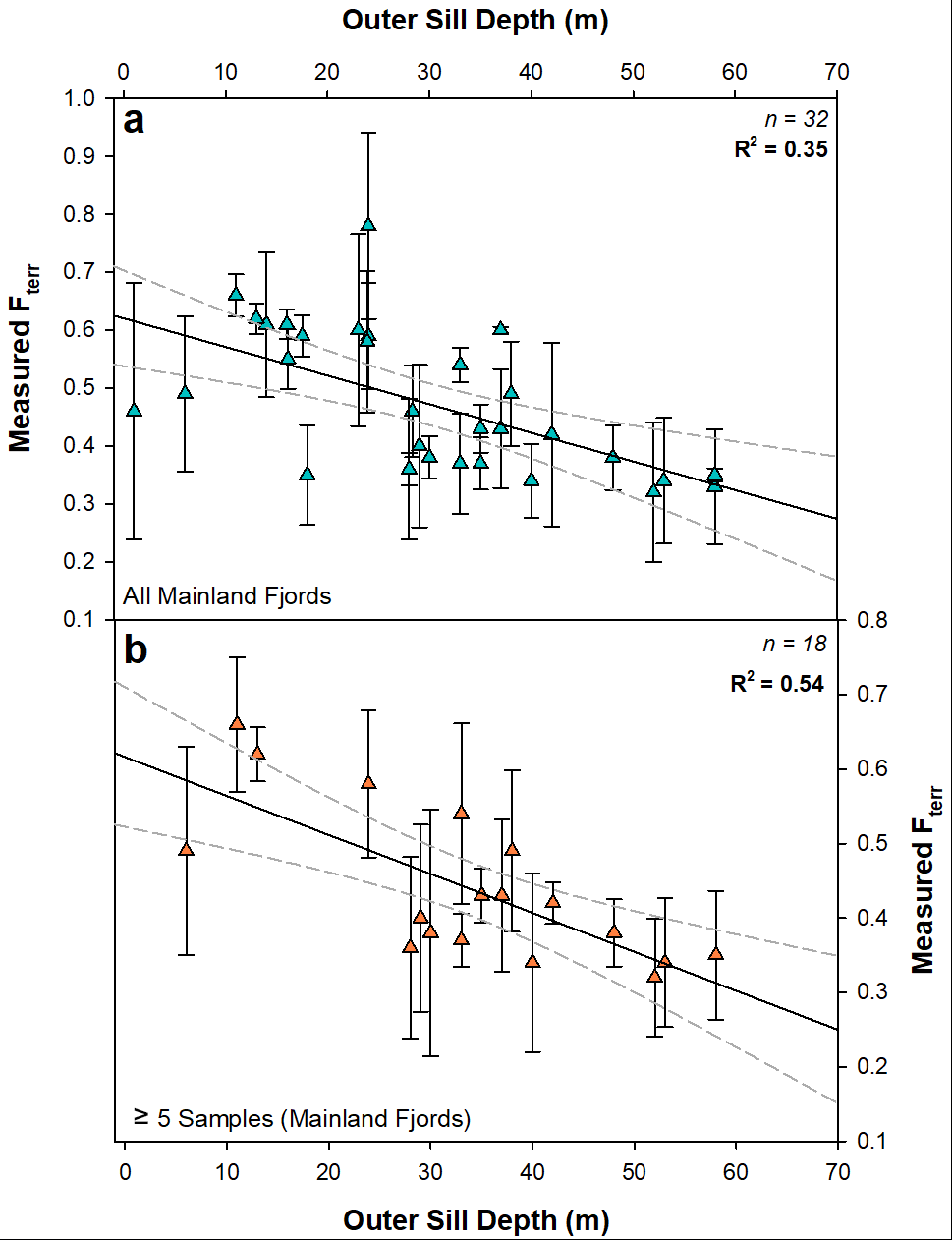


**Supplementary Figure 10.** Comparison of mainland fjord characteristics (Outer sill depth) and the fraction of OC derived from the terrestrial environment. (**a**) F_terr_ vs outer sill depth (m) (*n=32*); (**b**) F_terr_ vs outer sill depth (m) for the mainland fjords with ≥ 5 samples (*n = 18*). Dotted lines represent 95^th^ percentile confidence intervals. F_terr_= (-0.007 ×Outer Sill Depth) + 0.6823


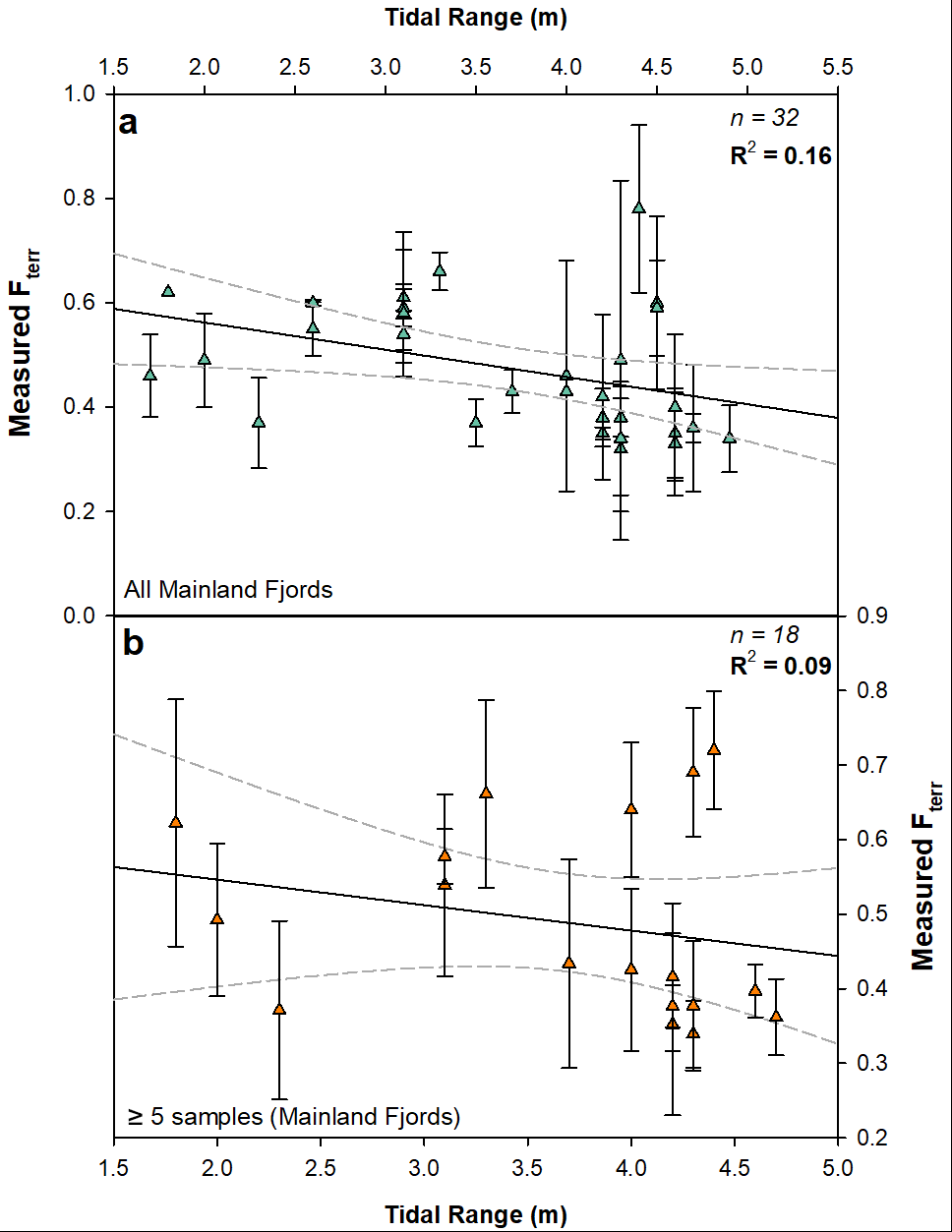


**Supplementary Figure 11.** The relationship between the fraction of OC that originates from the terrestrial environment and the tidal range. (**a**) All Scottish fjords (*n = 32*) (**b**) only fjords with ≥ 5 samples (*n = 18*). Dotted lines represent 95^th^ percentile confidence intervals. F_terr_= (-0.0645 × Tidal Range) + 0.7328


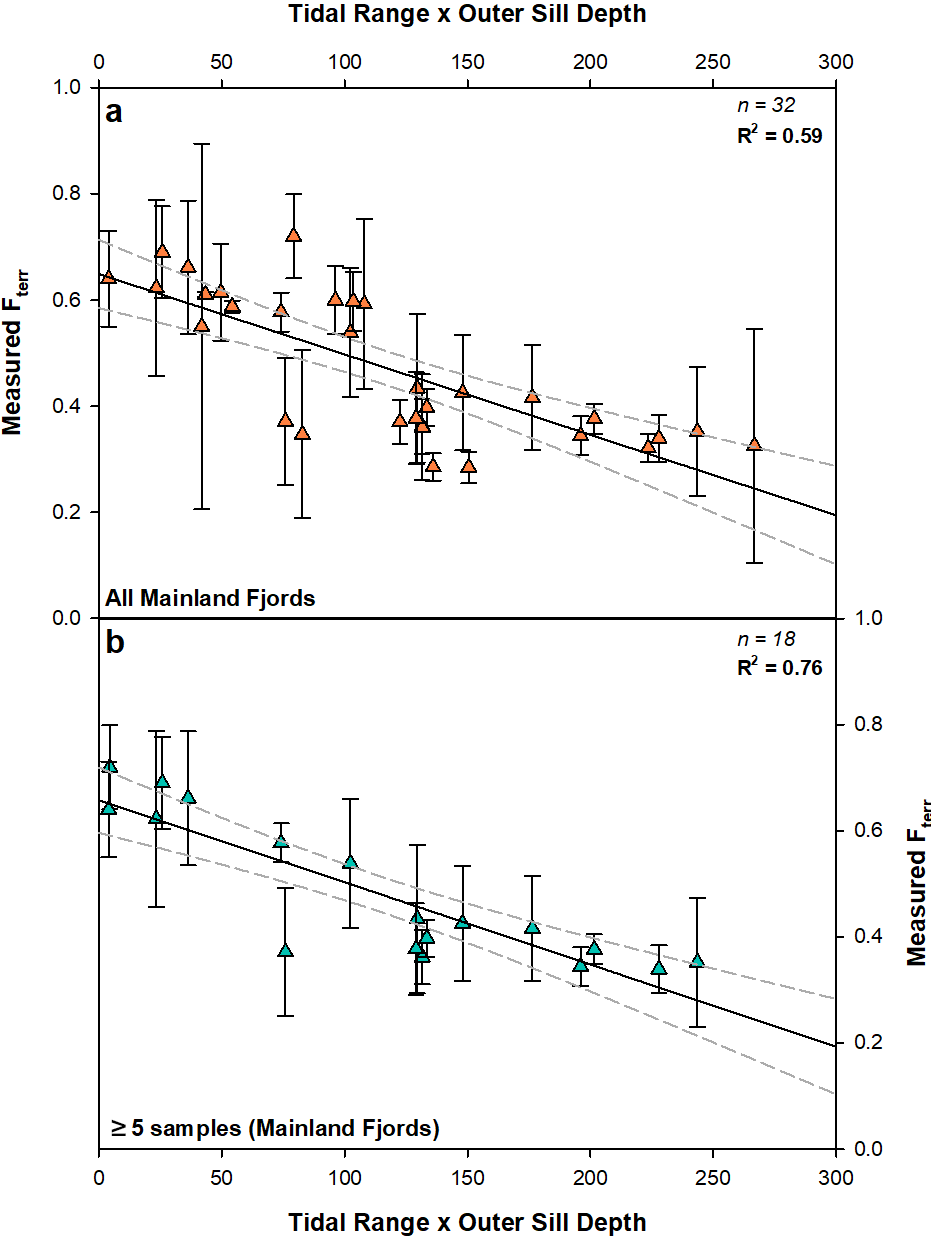


**Supplementary Figure 12.** Comparison of mainland fjord characteristics (tidal range x outer sill depth) and the fraction of OC derived from the terrestrial environment. (**a**) F_terr_ vs tidal range x outer sill depth (m) (*n=32*); (**b**) F_terr_ vs tidal range x outer sill depth (m) for the mainland fjords with ≥ 5 samples (*n = 18*). Dotted lines represent 95^th^ percentile confidence intervals. F_terr_ = (-0.0015 x (tidal range (m) x Outer Sill Depth (m)))+0.6534


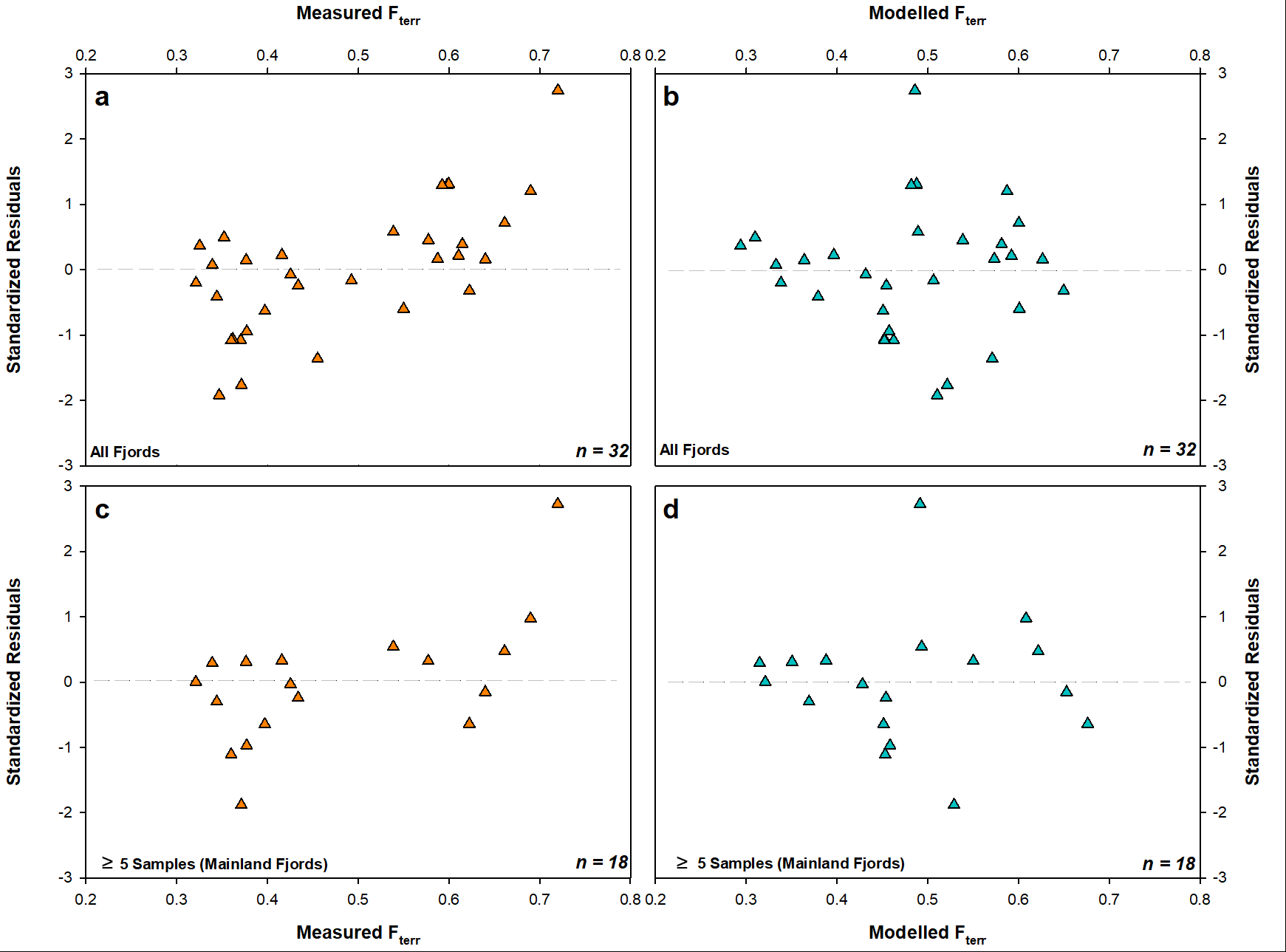


**Supplementary Figure 13.** Comparison of the standardized residuals to the measured and modelled F_terr_ values produced from the partial least squares regression (PLS). (**a-b**) all fjords. (**c-d**) Mainland fjords with ≥ 5 samples.

**
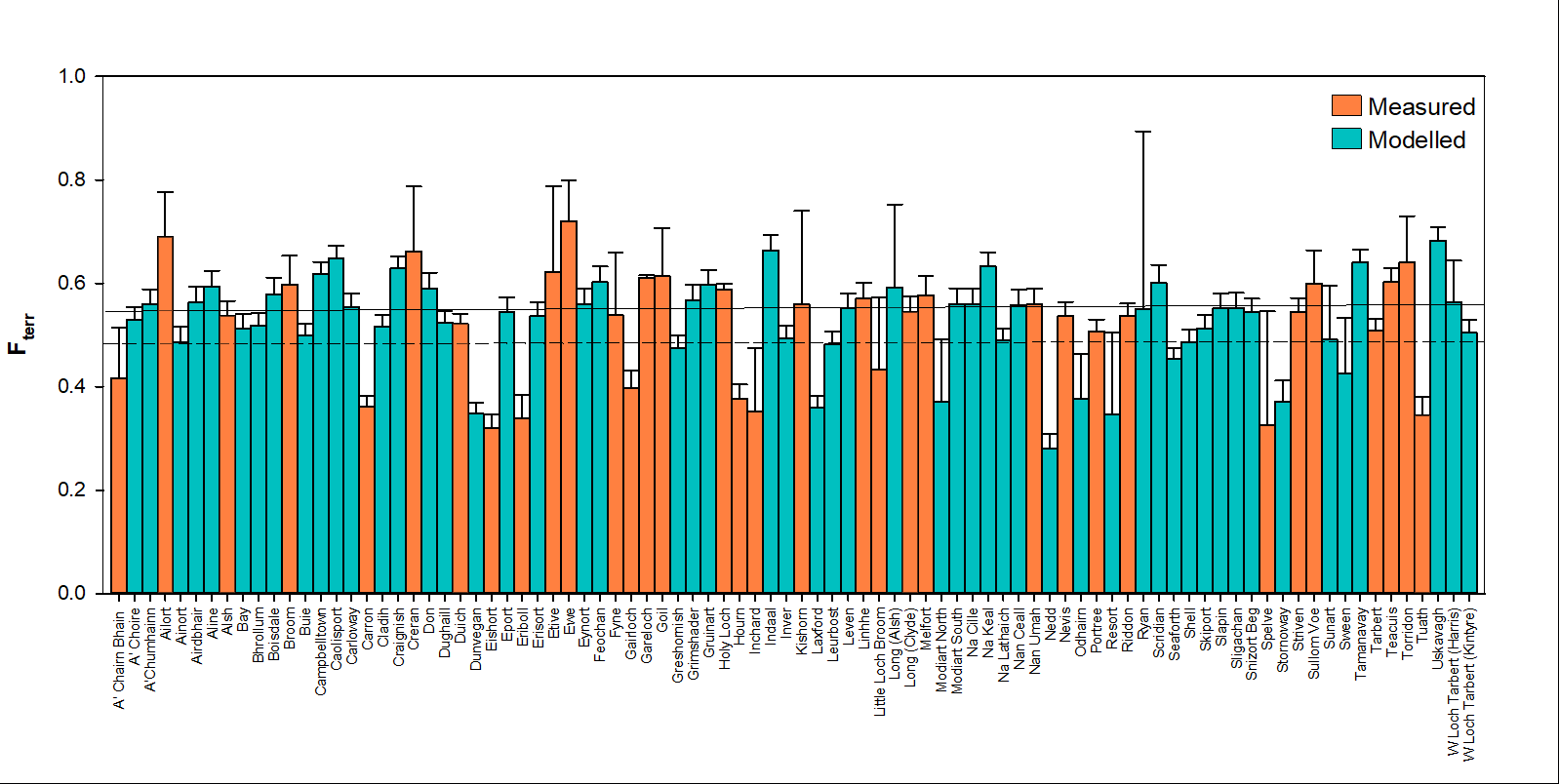
**

**Supplementary Figure 14**. Estimated F_terr_ values for the mid-latitude fjords of Scotland. Measured estimates are calculated from empirical data produced as part of this study. The modelled estimates are calculated using the relationship between F_terr_ , tidal range and outer sill depth (*Eq.4*). The dashed line indicates the mean F_terr_ value (0.47 ± 0.12) for the sites with empirical data, the solid line highlights the mean F_terr_ value (0.52 ± 0.10) for the complete dataset.


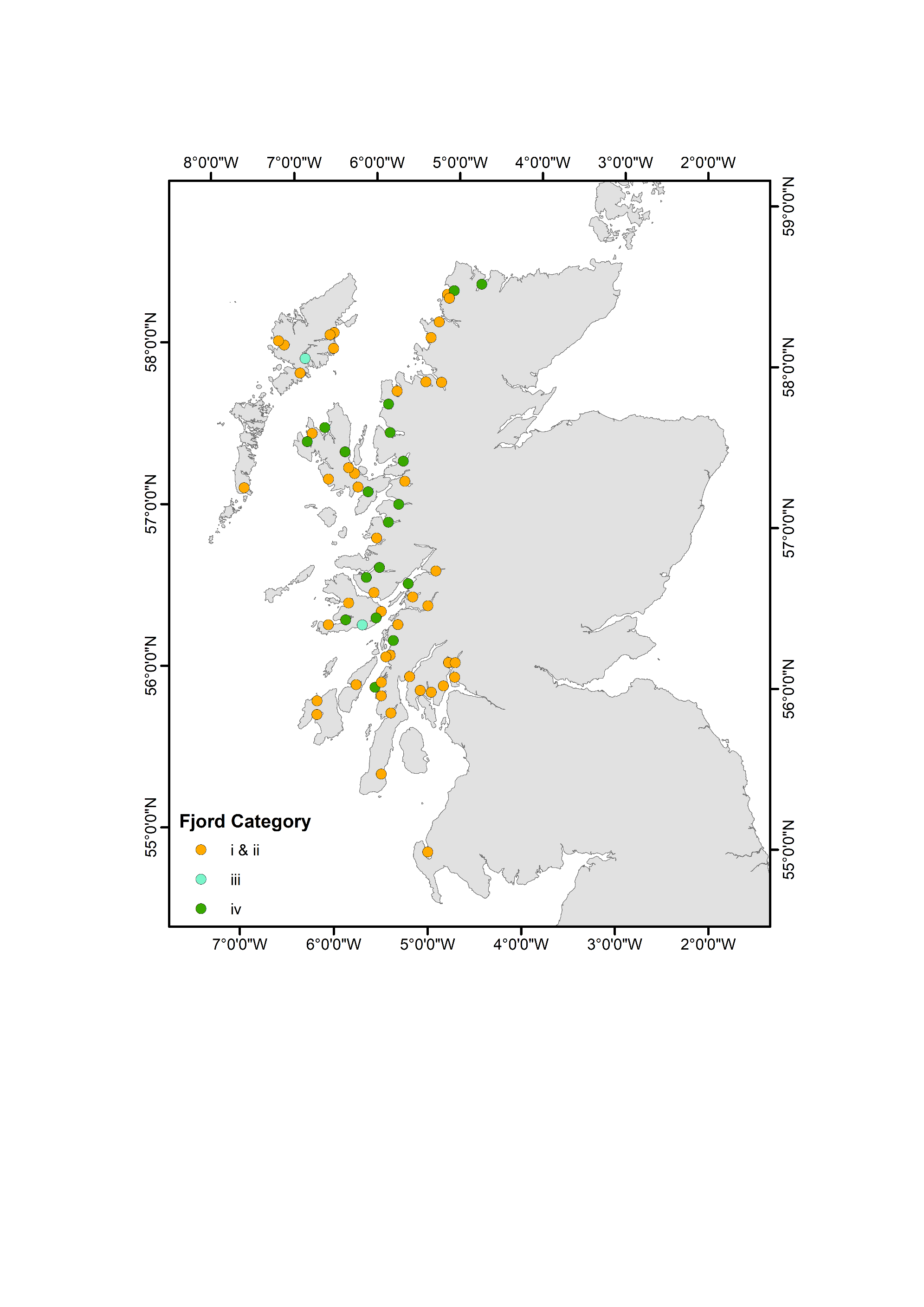


**Supplementary Figure 15.** Mainland Scottish fjords classified following the Faust and Knies, (2019) scheme (*see section 3.4*)

|  | **Inner Basin** | | **Main Basin** | | **Outer Basin** | |
| --- | --- | --- | --- | --- | --- | --- |
| **Fjord** | **Sill Depth (m)** | **F_terr_** | **Sill Depth (m)** | **F_terr_** | **Sill Depth (m)** | **F_terr_** |
| a' Chàirn Bhàin (Glencoul) | 25 | 0.44 ± 0.03 |  |  | 42 | 0.33 ± 0.03 |
| a' Chàirn Bhàin (Glendhu) | 20 | 0.52 ± 0.09 |  |  |  |  |
| Ailort | 4 | 0.53 ± 0.11 | 5 | 0.44 ± 0.02 | 11 | 0.55 ± 0.09 |
| Broom | 16 | 0.62 ± 0.06 |  |  | 13 | 0.56 ± 0.35 |
| Carron/Kishorn | 18 | 0.42 ± 0.02 |  |  | 28 | 0.33 ± 0.02 |
| Creran | 3 | 0.69 ± 0.13 |  |  | 11 | 0.65 ± 0.12 |
| Etive | 9 | 0.65 ± 0.16 |  |  | 13 | 0.40 ± 0.14 |
| Fyne | 16 | 0.62 ± 0.12 | 20 | 0.48 ± 0.06 | 33 | 0.45 ± 0.06 |
| Inchard | 23 | 0.43 ± 0.01 |  |  | 58 | 0.20 ± 0.06 |
| Linnhe | 9 | 0.67 ± 0.11 |  |  | 35 | 0.37 ± 0.06 |
| Little Loch Broom | 24 | 0.69 ± 0.19 |  |  | 26 | 0.49 ± 0.02 |
| Long | 25 | 0.61 ± 0.02 |  |  | 24 | 0.55 ± 0.01 |
| Sunart | 6 | 0.50 ± 0.12 | 31 | 0.43 ± 0.10 | 37 | 0.35 ± 0.06 |
| Teacuis | 12 | 0.46 ± 0.06 |  |  | 1 | 0.48 ± 0.14 |
| Torridon | 20 | 0.38 ± 0.03 | 53 | 0.37 ± 0.01 | 40 | 0.33 ± 0.02 |

**Supplementary Table 1.**  Mean F_terr_ of the surficial sediments in each of the fjord basins in this study.

| **Variable** | **Minimum** | **Maximum** | **Mean** | **Std. deviation** |
| --- | --- | --- | --- | --- |
| Loch Length | 3.00 | 60.50 | 14.22 | 11.79 |
| Tidal Range | 1.10 | 4.90 | 3.62 | 1.02 |
| Maximum Depth | 31.00 | 185.00 | 81.70 | 44.20 |
| High Water Area | 3.20 | 183.70 | 24.25 | 33.09 |
| Low Water Area | 1.60 | 175.50 | 22.44 | 31.65 |
| Watershed Area | 12.00 | 1820.00 | 257.15 | 387.22 |
| Annual Rainfall | 1100.00 | 2500.00 | 1922.73 | 359.71 |
| Runoff | 10.10 | 3549.00 | 468.46 | 776.67 |
| Kinetic Energy | 0.00 | 7521.30 | 641.43 | 1780.89 |
| Mean Depth | 7.50 | 59.50 | 27.35 | 14.45 |
| Fresh/Tide Ratio | 1.20 | 120.40 | 17.28 | 24.16 |
| Number of sills | 0.00 | 6.00 | 2.09 | 1.88 |
| Outer Sill Depth | 1.00 | 58.00 | 29.17 | 14.61 |
| Tidal Range x Outer Sill Depth | 4.00 | 266.80 | 109.90 | 70.27 |
| F_terr_ | 0.32 | 0.78 | 0.48 | 0.12 |

**Supplementary Table 2.** Summary statistics for the environmental variables from the 32 fjords.

|  | **F_terr_** | | | | |
| --- | --- | --- | --- | --- | --- |
| **Variable** | **Correlation Matrix (*Pearson*)** | **Confidence intervals (95%) / Lower bound** | **Confidence intervals (95%) / Upper bound** | **Coefficients of determination (*Pearson*)** | **p-Values**  **(*Pearson*)** |
| Loch Length | 0.107 | -0.245 | 0.434 | 0.011 | 0.554 |
| Tidal Range | -0.418 | -0.666 | -0.087 | 0.174 | 0.016 |
| Maximum Depth | -0.059 | -0.394 | 0.290 | 0.003 | 0.745 |
| High Water Area | 0.086 | -0.265 | 0.417 | 0.007 | 0.636 |
| Low Water Area | 0.091 | -0.260 | 0.421 | 0.008 | 0.614 |
| Watershed Area | 0.185 | -0.169 | 0.496 | 0.034 | 0.304 |
| Annual Rainfall | -0.045 | -0.382 | 0.303 | 0.002 | 0.805 |
| Runoff | 0.167 | -0.187 | 0.483 | 0.028 | 0.352 |
| Kinetic Energy | 0.087 | -0.264 | 0.418 | 0.008 | 0.629 |
| Mean Depth | -0.057 | -0.393 | 0.292 | 0.003 | 0.753 |
| Fresh/Tide Ratio | 0.229 | -0.124 | 0.531 | 0.053 | 0.199 |
| Number of sills | -0.008 | -0.351 | 0.336 | 0.000 | 0.963 |
| Outer Sill Depth | -0.603 | -0.784 | -0.327 | 0.363 | 0.000 |
| Tidal Range x Outer Sill Depth | -0.642 | -0.807 | -0.383 | 0.412 | 0.000 |
| F_terr_ | 1 | 1 | 1 | 1 | 0 |

**Supplementary Table 3.** Correlation and covariance tests between F_terr_ and fjord variables.

|  | **Fraction Terrestrial** | | **Surficial Sediment OC_terr_ Stock** | | **OC_terr_ Density** | |
| --- | --- | --- | --- | --- | --- | --- |
| **Rank** | **Fjord** | **F_terr_** | **Fjord** | **tonnes** | **Fjord** | **tonnes km^-2^** |
| 1 | Ewe | 0.72 | Fyne | 246457 | Craignish | 2181 |
| 2 | Ailort | 0.69 | Linnhe | 237525 | Feochan | 1986 |
| 3 | Tuath | 0.68 | Snizort | 87590 | Campbeltown | 1919 |
| 4 | Indaal | 0.66 | Ewe | 58331 | Sween | 1688 |
| 5 | Creran | 0.66 | Ryan | 55948 | Gare Loch | 1577 |
| 6 | West Loch Tarbert | 0.66 | Long | 49337 | Little Loch Broom | 1553 |
| 7 | Caolisport | 0.65 | Toridon/Sheildaig | 42990 | Euphort | 1503 |
| 8 | Sween | 0.64 | Sunart/Tecuis | 41312 | Long | 1468 |
| 9 | Teacuis | 0.64 | Craignish | 39265 | Fyne | 1404 |
| 10 | Na Cille | 0.63 | Etive | 37235 | Ryan | 1388 |
| 11 | Craignish | 0.63 | Tuath | 34735 | Leven | 1378 |
| 12 | Etive | 0.62 | Duich/Alsh/Long | 34057 | Etive | 1344 |
| 13 | Campbelltown | 0.62 | Scridian | 33590 | Scridian | 1328 |
| 14 | Goil | 0.61 | Na Keal | 33258 | Riddon/Kyles of Bute | 1326 |
| 15 | Gareloch | 0.61 | Little Loch Broom | 31687 | Ewe | 1314 |
| 16 | Feochan | 0.60 | Sween | 30720 | Caolisport | 1313 |
| 17 | Tarbert | 0.60 | Indall | 27542 | Striven | 1273 |
| 18 | Ryan | 0.60 | Hourn | 27207 | Airlort | 1216 |
| 19 | Striven | 0.60 | Carron/Kishorn | 20349 | Moidart | 1175 |
| 20 | Broom | 0.60 | Eriboll | 20304 | Clash | 1175 |
| 21 | Gruinart | 0.60 | Nevis | 20167 | Luirboist | 1165 |
| 22 | Aline | 0.59 | Dunvegan | 18716 | Eireasort | 1152 |
| 23 | Little Loch Broom | 0.59 | Gare Loch | 18292 | Holy Loch | 1139 |
| 24 | Don | 0.59 | Sullom | 18012 | Indall | 1138 |
| 25 | Holy Loch | 0.59 | Broom | 17510 | Nedd | 1137 |
| 26 | Boisdale | 0.58 | Bay | 17334 | Creran | 1122 |
| 27 | Long (Clyde) | 0.58 | Caolisport | 16416 | Bay | 1111 |
| 28 | Leven | 0.57 | Striven | 15663 | Na Keal | 1087 |
| 29 | Grimshader | 0.57 | Creran | 14921 | Tuath | 1062 |
| 30 | Airdbhair | 0.56 | Eireasort | 13706 | Spelve | 1060 |
| 31 | Uskavagh | 0.56 | Leven | 10334 | Laxford | 1048 |
| 32 | Nan Ceall | 0.56 | Buie | 10275 | Broom | 1042 |
| 33 | Modiart South | 0.56 | West Loch Tarbert | 10235 | Airnort | 1039 |
| 34 | Modiart North | 0.56 | Spelve | 9328 | Portree | 1036 |
| 35 | Kentra Bay | 0.56 | Gairloch | 8786 | Sullom | 995 |
| 36 | Eynort | 0.56 | Laxford | 8696 | Buie | 969 |
| 37 | A'Chumhainn | 0.56 | Seaforth/Shiport | 8494 | Slapin/Eishort | 966 |
| 38 | Na Lathaich | 0.56 | Euphort | 8463 | Linnhe | 938 |
| 39 | Carloway | 0.55 | Tarbert (Jura) | 8386 | Griomsiadair | 923 |
| 40 | Slapin | 0.55 | a Chairn Bhain/ | 7930 | Inchard | 919 |
| 41 | Skiport | 0.55 | Airlort | 7539 | West Loch Tarbert | 890 |
| 42 | Leurbost | 0.55 | Campbeltown | 6909 | Tarbert (Jura) | 883 |
| 43 | Riddon | 0.55 | Melfort | 6620 | a Choire | 842 |
| 44 | Sligachan | 0.54 | Goil | 6204 | Duich/Alsh/Long | 839 |
| 45 | Long (Alsh) | 0.54 | Feochan | 5362 | Aline | 839 |
| 46 | Stornoway | 0.54 | nan Uamh | 5119 | na Cille | 835 |
| 47 | Eport | 0.54 | Boisdale/Baghasdail | 4810 | Sunart/Tecuis | 835 |
| 48 | Fyne | 0.54 | Holy Loch | 3873 | Don | 810 |
| 49 | Erisort | 0.54 | Slapin/Eishort | 3863 | Hourn | 807 |
| 50 | Nedd | 0.54 | Inchard | 3677 | Sligachan | 782 |

**Supplementary Table 4.** Mainland fjords ranked by F_terr,_ OC_terr_ stock (tonnes) and OC_terr_ density (tonnes km^-2^).

|  | **Fraction Terrestrial** | | **Surficial Sediment OC_terr_ Stock** | | **OC_terr_ Density** | |
| --- | --- | --- | --- | --- | --- | --- |
| **Rank** | **Fjord** | **F_terr_** | **Fjord** | **tonnes** | **Fjord** | **tonnes km^-2^** |
| 1 | Stromness Voe | 0.66 | Sandsound | 8989 | Dales North/Colla | 1861 |
| 2 | Sandsound Voe | 0.64 | Ronas | 7603 | Ura | 1786 |
| 3 | Balta Harbour | 0.63 | Olna/Busta/Airth | 7341 | Sandsound | 1474 |
| 4 | Whitness Voe | 0.63 | Clift/Lang | 7078 | Ronas | 1246 |
| 5 | Mid Yell | 0.63 | Dales North/Colla Firth | 6884 | Gluss | 1229 |
| 6 | Ura Firth | 0.62 | Weisdale | 4917 | Balta | 1144 |
| 7 | Grunting Voe | 0.62 | Ura | 3215 | Weisdale | 1069 |
| 8 | Whale Firth | 0.62 | Whiteness | 3112 | Whiteness | 1037 |
| 9 | Weisdale Vow | 0.62 | Basta N | 2825 | Clift/Lang | 851 |
| 10 | Cat Firth | 0.62 | Bast/Pund | 1986 | Stromness | 768 |
| 11 | Lax Firth | 0.62 | Balta | 1716 | Olna/Busta/Airth | 734 |
| 12 | Vidlin Voe | 0.62 | Dales South | 1585 | Basta N | 689 |
| 13 | Colla Firth | 0.61 | Whale | 1443 | Bast/Pund | 685 |
| 14 | Dales Voe North | 0.60 | Valia/Grunting/Sell | 1396 | Valia/Grunting/Sell | 537 |
| 15 | Swinning Voe | 0.60 | Gluss | 1352 | Dales South | 528 |
| 16 | Dale Voe South | 0.60 | Stromness | 691 | Whale | 465 |
| 17 | Busta Voe | 0.60 |  |  |  |  |
| 18 | Gluss Voe | 0.60 |  |  |  |  |
| 19 | Ronas Voe | 0.59 |  |  |  |  |
| 20 | Burra Firth | 0.59 |  |  |  |  |
| 21 | Aith Voe | 0.58 |  |  |  |  |
| 22 | Olna Firth | 0.58 |  |  |  |  |
| 23 | Basta Voe | 0.46 |  |  |  |  |
| 24 | Clift Sound | 0.37 |  |  |  |  |
| 25 | Vaila Sound | 0.36 |  |  |  |  |

**Supplementary Table 5.** Fjards (Shetland Islands) ranked by F_terr,_ OC_terr_ stock (tonnes) and OC_terr_ density (tonnes km^-2^).

|  | **OC Density (tonnes km^-2^)** | | | | |
| --- | --- | --- | --- | --- | --- |
| **Fjord Cat** | **Mean** | **Std. Dev** | **Median** | **Min** | **Max** |
| i and ii | 1811.18 | 655.38 | 1746.55 | 816.20 | 3468.83 |
| iii | 930.30 | 56.09 | 930.65 | 899.99 | 969.30 |
| iv | 2056.03 | 549.18 | 1968.06 | 858.27 | 2987.00 |

**Supplementary Table 6.** Surficial (top 10cm) sediment OC storage across Scotland’s fjords characterized following the Faust and Knies, (2019) scheme (*see section 3.4*).

**References (*all included in main text*)**

Edwards, A., Sharples, F., 1986. Scottish sea lochs: a catalogue. Scottish Marine Biological Association.

Faust, J.C., Knies, J., 2019. Organic Matter Sources in North Atlantic Fjord Sediments. Geochemistry, Geophysics, Geosystems, 20, 2872–2885. <https://doi.org/10.1029/2019GC00838>

Smeaton, C., Austin, W.E.N., 2017. Sources, Sinks, and Subsidies: Terrestrial Carbon Storage in Mid-latitude Fjords. J. Geophys. Res. Biogeosciences, 122, 2754–2768. <https://doi.org/10.1002/2017JG003952>

Smeaton, C., Austin, W.E.N., 2019. Where’s the Carbon : Exploring the Spatial Heterogeneity of Sedimentary Carbon in Mid-Latitude Fjords. Frontiers in Earth Science, 7, 1–16. <https://doi.org/10.3389/feart.2019.00269>

Smeaton, Craig, Hunt, C.A., Turrell, W.R., Austin, W.E.N., 2021b. Marine Sedimentary Carbon Stocks of the United Kingdom’s Exclusive Economic Zone. Frontiers in Earth Science, 9. <https://doi.org/10.3389/feart.2021.593324>

Smeaton, C., Miller, L.C., Ladd, C.J.T., O'Dell, A., Austin, W.E.N. ,2022. Bulk elemental and stable isotope composition of organic matter from terrestrial, intertidal, and marine environments, UK, 2016-2021. NERC Environmental Information Data Centre. <https://doi.org/10.5285/a445a7a8-528d-4e0b-9094-28cbcd449367>
